# Nutritional psychiatry research: an emerging discipline and its intersection with global urbanization, environmental challenges and the evolutionary mismatch

**DOI:** 10.1186/1880-6805-33-22

**Published:** 2014-07-24

**Authors:** Alan C Logan, Felice N Jacka

**Affiliations:** 1 CAMNR, 23679 Calabasas Road Suite 542, Calabasas, CA 91302, USA; 2 School of Medicine, Deakin University, IMPACT SRC, PO Box 281, Geelong, VIC 3220, Australia

## Abstract

In 21st-century public health, rapid urbanization and mental health disorders are a growing global concern. The relationship between diet, brain function and the risk of mental disorders has been the subject of intense research in recent years. In this review, we examine some of the potential socioeconomic and environmental challenges detracting from the traditional dietary patterns that might otherwise support positive mental health. In the context of urban expansion, climate change, cultural and technological changes and the global industrialization and ultraprocessing of food, findings related to nutrition and mental health are connected to some of the most pressing issues of our time. The research is also of relevance to matters of biophysiological anthropology. We explore some aspects of a potential evolutionary mismatch between our ancestral past (Paleolithic, Neolithic) and the contemporary nutritional environment. Changes related to dietary acid load, advanced glycation end products and microbiota (via dietary choices and cooking practices) may be of relevance to depression, anxiety and other mental disorders. In particular, the results of emerging studies demonstrate the importance of prenatal and early childhood dietary practices within the developmental origins of health and disease concept. There is still much work to be done before these population studies and their mirrored advances in bench research can provide translation to clinical medicine and public health policy. However, the clear message is that in the midst of a looming global epidemic, we ignore nutrition at our peril.

## Introduction

*Urbanization*, commonly defined as the change in size, density and heterogeneity of cities, is a rapidly occurring global phenomenon. Projections suggest that within the next several decades, urbanization will continue in both developed and developing nations, such that by 2050 86% of the population of developed nations and 64% of developing nations will be urban residents. More immediately, by 2030, it is estimated that there will be an additional 1.35 billion people living in world cities [1,2].

*Urbanicity*, a term that more specifically refers to the presence of conditions that are particular to urban vs nonurban areas, is ultimately defined as the impact of living in urban areas at a given time [3]. We acknowledge that urbanization and urbanicity can bring many potential benefits, including, but not limited to, those involving commerce, trade, education, communications and the efficient delivery of government and health-care services; however, the problems associated with rapid urbanization are many. In particular, urbanization is increasingly linked to changes in behavior that contribute to noncommunicable diseases (NCDs) or so-called diseases of civilization. For example, urbanization has been associated with diminished levels of physical activity, compromised sleep and unhealthy dietary choices [3-8].

A related issue is the globalization of the food industry, reflected in substantial changes in efficiencies of production, marketing, transport, advertising and sale of food; this has prompted profound shifts in the composition of diets globally and has been one of the major drivers of changes in the distribution and burden of the NCDs over the second half of the 20th century [9,10]. These changes may particularly affect those residing in deprived urban and suburban areas where there is a far higher density of retail venues that facilitate the consumption of unhealthy food commodities [11].

Among the NCDs, mental disorders—major depression and anxiety in particular—have been described as an impending global epidemic. Although less is known concerning the prevalence of these disorders in developing nations than elsewhere [12,13], all data indicate that the burden of disease attributable to mental disorders will continue to rise globally over the coming decades [14]. Living in an urban environment [15-17], as well as global shifts associated with urbanicity [18-21], the food supply and food industry [22], climate change [23], food insecurity [24,25], overnutrition and underactivity [26] and an overall transition from traditional lifestyles [27-29] have each been linked to increases in depression and other mental disorders. It is understood that depression and other mental disorders are not exclusive to urban areas; however, higher rates in urban areas have been reported with a degree of consistency [30,31]. Moreover, looking specifically at neighborhood disadvantage, the adjusted odds of experiencing an emotional disorder are 59% higher in urban youth vs those in rural areas [32].

Regardless of the urban–rural divide, within developed nations, up to one-third of all visits to primary care clinicians involve patients with emotional disorders, most notably anxiety and/or depressive conditions [33]. In both developed nations and those in the midst of an epidemiological transition, depression is rapidly rising within the burden of disease rankings [34-36]. Moreover, depression is already part of an epidemic of comorbidity and will likely become even more so. Specifically, obesity, type 2 diabetes and other chronic diseases strongly associated with lifestyle behaviors are highly comorbid with depression [37,38].

Because diagnosable depression and anxiety [39-41] (and their subclinical and subthreshold variants that may otherwise escape detection [42]) are also outcomes associated with, and drivers of, subsequent cardiovascular and other chronic disease states (that is, they are both upstream and downstream of “physical” illness), a multidisciplinary approach to prevention and treatment is necessary [43]. The results of studies involving adults with depression indicate that treatment outcomes from standard first-line antidepressant or psychotherapy interventions (for example, cognitive-behavioral therapy) are far from being universally adequate. Notwithstanding the debates concerning the placebo effect vs antidepressant efficacy [44,45] and their potential adverse effects [46] (as well as publication biases concerning cognitive–behavioral therapy [47-49]), treatment resistance, relapse and nonresponse remain a reality [50,51]. Moreover, even with optimal care and access to services, the results of modeling studies suggest that only a proportion of the burden of mental disorders can be averted, pointing to a clear need to focus on prevention to reduce the incidence of mental disorders [52]. From a research perspective, there is an urgent need to examine various environmental or lifestyle variables more thoroughly, including those wherein there are scientifically plausible mechanisms that can explain risk.

With this background in mind, we explore recent developments within an emerging discipline known as nutritional psychiatry. The relationship between dietary patterns and risk of mental health disorders has been the subject of intense research in recent years. There seems little doubt that dietary patterns and mental health, as well as other NCDs, are intertwined with socioeconomic circumstances [53]. We examine some of the potential socioeconomic and environmental challenges that detract from a dietary pattern or optimal nutrient intake that might otherwise support positive mental health.

As discussed below, in the context of urbanization, cultural and technological changes and the global industrialization and ultraprocessing of food, the findings related to nutrition and mental health are connected to some of the most pressing issues of our time. They are also of relevance to matters of biophysiological anthropology and a potential evolutionary mismatch between our ancestral past and the contemporary nutritional environment. At the outset, we acknowledge that much of our focus here is on depression. This is largely because depression has received the lion’s share of international research attention as it relates to some of the nutritional and environmental variables; however, it should be understood that these discussions are of relevance to diverse topics ranging from reductions in violent behavior [54] to overall positive mental outlook in those who are otherwise well-adjusted [55,56].

### Nutritional psychiatry research

Among the variables that might afford resiliency against mental disorders or, on the other hand, contribute to risk, diet has emerged as a strong candidate [57]. Viewed superficially, nutrition is a variable that needs little explanation in terms of its mechanistic potential. The brain operates at a very high metabolic rate, utilizing a substantial proportion of the body’s energy and nutrient intake. The very structure and function (intra- and intercellular communication) of the brain is dependent upon amino acids, fats, vitamins and minerals and trace elements. The antioxidant defense system operates with the support of nutrient cofactors and phytochemicals and is of particular relevance to psychiatry [58]. Similarly, the functioning of the immune system is of substantial importance to psychiatric disorders and profoundly influenced by diet and other lifestyle factors [59]. Neuronal development and repair mechanisms (neurotrophic factors) throughout life are also highly influenced by nutritional factors [60]. In short, a theoretical framework of biological mechanisms whereby nutrition could exert its influence along a continuum ranging from general mental well-being to neuropsychiatric disorders is highly plausible. However, despite its role as the foundation of physiological processes, nutrition as a factor in promoting positive mental health (or working against it) has traditionally suffered from scientific neglect and/or poorly designed studies that compromise interpretation.

Encouragingly, there are signposts indicating a major shift related to nutritional matters in mental health. Given the aforementioned background on the burden of mental disorders, this development is timely. A variety of epidemiological studies, including high-quality prospective studies, have linked adherence to healthful dietary patterns with lowered risk of anxiety and/or depression [61-67]. The results of these studies indicate that nutrition may provide a very meaningful layer of resiliency. The effect sizes suggest a clinically relevant value, not simply a minimal statistical significance. Healthy dietary patterns characterized by a high intake of vegetables, fruits, potatoes, soy products, mushrooms, seaweed and fish have recently been associated with a decreased risk of suicide [68]. Specific elements of the diet—green tea and coffee, for example—have also been linked to a decreased risk of depressive symptoms [69]. Moreover, nutrition is emerging as a critical factor within the developmental origins of health and disease (DOHaD) construct. Early nutrition is now being linked, convincingly, to later mental health outcomes [70]. Moreover, at the other end of the lifespan, adherence to a Mediterranean diet results in better cognitive outcomes and a reduced risk for dementia [71,72].

In support of these large population studies, at the opposite end of the research continuum, sophisticated bench studies are determining the pathways whereby individual elements of diet—polyphenols, for example—can influence brain function [73-75]. The area of dietary fats is yet another area of intense scrutiny, one where high-fat, obesogenic diets consumed during the pre- and perinatal period have been associated with long-term changes in neurotransmission, brain plasticity and behavior in offspring [76-78]. Moreover, beyond the detrimental effects of excessive fat *per se*, recent experimental evidence suggests that all fats, and even classes of fats (for example, various saturated fat sources), cannot be painted with the same brush in the context of brain health [79-82]. It is also worth noting that the results of numerous international studies have linked low blood cholesterol levels with suicide risk [83], a finding that might be related to low levels of the more specific high-density lipoprotein that is otherwise promoted through healthy dietary practices [84].

On the one hand, in between the epidemiological and experimental evidence, the results of a variety of clinical studies suggest the value of ω-3-rich oils in disorders including, but not limited to, bipolar depression [85], posttraumatic stress disorder [86], major depression [87] and the indicated prevention of psychosis [88]. On the other hand, given the brain’s continuous use of such a wide variety of nutrients for its structure and function, and the potential errors in metabolism that may be at play in mental disorders, the traditional focus on a single-nutrient resolution [89] may mask the potential value of multinutrient interventions [90]. Zinc provides a good example. The results of a number of preclinical and clinical studies have indicated that zinc supplementation may be helpful for people with major depression [91], and low dietary zinc has not only been correlated with depression [92], but has also been linked to abnormalities in the response to antidepressant medication [93]. However, zinc is well-established to play key roles in the metabolism of ω-3 and other essential fatty acids [94]. Indeed, there are complex ways in which ω-3 and zinc work together in the epigenetic control of human neuronal cells [95]. It is likely, therefore, that in the clinical setting, a combination of nutrients may provide better outcomes [96]. In the process of translational medicine, further examination of multinutrient bench and intervention studies will be necessary [97,98].

For better or for worse, large percentages of adults in developed nations are consumers of dietary supplements [99], and this seems especially true of those with mental disorders. For example, two-thirds of all patients with mood disorders in one recent Canadian study were found to be supplement consumers, and 58% were taking supplements in combination with psychotropic medications [100]. Such findings only add to the urgency of evaluating the role and importance of nutritional variables, ranging from overall dietary patterns to the epigenetic controls exerted by isolated nutrients. Moreover, there is a clear need to explicate in detail the ways in which dietary supplements may interact with medications, particularly the widely prescribed classes of antidepressants and anxiolytics.

There is also an imperative to examine more closely the ways in which the nonnutritional elements of the modern diet may contribute to various brain-related disorders. Such research should include, but is certainly not limited to, the possible role of food-derived residual pesticides in brain health [101] and the ways in which synthetic food dyes, artificial sweeteners and flavor enhancers may influence behavior [102-104]. The results of preliminary randomized, placebo-controlled research suggest that short-term exposure to gluten can induce depressive symptoms in people with nonceliac gluten sensitivity [105].

Although it is commonly held that there is “no health without mental health” [106], the emerging evidence suggests that the opposite is also true: Good physical health and aerobic fitness are vitally important to mental health [107,108]. We would argue that if a healthy diet influences mood, it might also increase the likelihood that an individual will remain physically active. This might be especially true in people with depression or obesity, in whom mood-related barriers diminish the desire perform physical activity or minimize its postexertion appeal [109-111]. A positive emotional state encourages future participation in physical activity [112,113]. Supplementation of individual components of traditional diets, such as vitamin C, have been shown to reduce heart rate, perceived exertion and general fatigue postexercise in overweight adults [114]. Adoption of the Mediterranean diet for 10 days has been found to result in significantly increased vigor, alertness and contentment among participants vs controls [115]. Adherence to traditional diets, such as the Mediterranean diet, has been linked to objectively measured physical performance [116,117]. More research is required to tease apart the ways in which nutrition can be used to tackle the motivational barriers to initiating and maintaining a routine of physical activity.

In people with mental disorders (and even acute diminished mental outlook), there appears to be involvement of chronic low-grade inflammation [118], oxidative stress [119], impaired metabolism [120], alterations to microbiota [121] and numerous other pathways that can be mediated by nutrition. In particular, there is currently much enthusiasm surrounding the potential of probiotics and prebiotics (or any agents capable of causing favorable shifts in the intestinal microbiota) as a means to promote positive mental health [122]. However, there has been little attention paid to the central role of diet as a variable working for or against the potential value, particularly over the long term, of these so-called psychobiotics [123,124]. The same high-fat Westernized dietary factors that may negatively alter intestinal microbiota and promote intestinal permeability (provoking low-grade systemic inflammation via lipopolysaccharide endotoxin [121]) have also been shown to disrupt the critically important blood–brain barrier [125-127]. There may be untold consequences of diet-induced assaults to the integrity of both intestinal and the blood–brain barriers as they relate to mental health.

Clearly, there have been exciting advances in nutritional psychiatry research. The overall findings make it clear that nutrition matters in mental health. However, the degree to which nutrition matters remains unknown; the existing research, beyond validating nutrition as a variable of importance, has also served to generate a myriad of research questions that will require evidence-based answers. This in itself supports our call for broad collaboration to move this field of investigation forward and to explicate mechanistic pathways in order to identify targets for intervention. Many of the obstacles to learning how and the extent to which nutrition can work for or against positive mental health are related to the complex ways in which nutrition is intertwined with social, cultural and economic factors. In the next section, we address some of those complexities.

### Environmental challenges, social determinants

The available evidence suggests that traditional dietary habits are beneficial for positive mental health and that unhealthy dietary choices are a related (but independent) detractor from a state of good mental health. Assuming for a moment that the evidence continues to grow more robust (an assumption generally supported by current meta-analysis results [66,128]), the critical components of next-phase research will include further examination of the environmental factors that push toward (or pull away from) healthy, traditional dietary patterns. The environmental forces that facilitate a pull away from traditional diets appear to be extraordinarily strong.

Before examining some of the environmental factors that may work against nutrition for mental health, it is important to consider two contextual facts. First, mental health disparities within nations are commonplace; for example, depression does not occur randomly, and its rates, along with other mental disorders, are far higher in the disadvantaged social strata [129-131]. Second, the volumes of international research concerning obesity are likely of major relevance to the global mental health crisis. There seems little dispute that obesity can contribute to subsequent mental health problems. However, there are convincing prospective studies showing just the opposite—that depression and/or anxiety in children and adults predict subsequent weight gain over time [39,132-137]. Therefore, scientific discussions concerning environmental factors related to obesity are highly relevant to the topic of mental health [138]. Also, much like depression, obesity is not randomly distributed within nations; it, too, is slanted toward neighborhood deprivation and lower socioeconomic strata (SES) [139].

Global urbanization, highlighted in the Introduction, is clearly linked to higher consumption of energy-dense, nutrient-poor foods and beverages. The results of epidemiological and experimental research suggest that it is the highly palatable combination of sugar, fat and sodium that plays a key role in the attractiveness of such foods [140-142]. From an evolutionary perspective, the pleasure associated with energy-dense food consumption in the ancestral environment could motivate intake as a means of offsetting the scarcity of foods. Moreover, contemporary economic factors magnify the allure of inexpensive, widely available, energy-dense and nutrient-poor foods. Research indicates that higher costs are associated with nutrient-rich fruits and vegetables and quality protein sources such as fish and lean meat [143-145].

Animal experiments with the so-called cafeteria (Westernized) diet back up the contention that the availability and variety of palatable energy-dense foods drives long-term overconsumption of those foods, coincident with alterations to gene expression in the brain’s reward system [146,147]. Indeed, experimental models of early-life stress show that the cafeteria diet can dampen the stress response, indicating that its consumption may be a form of self-medication [148]. This is supported by human research on the relationship between psychological distress and the increased consumption of so-called comfort foods [149-151]. Indeed, research shows that, at least in healthy adults, the direct infusion of fatty acids into the stomach (bypassing visual, gustatory and olfactory cues) can rapidly attenuate a laboratory-induced negative mood state [152].

Taken together, the physiological responses to the consumption of energy-dense comfort foods are likely to be behaviorally reinforced in people with mental health disorders. If we factor in economics and convenience in people who experience financial stress and physical fatigue, the likelihood of these foods’ being preferentially consumed increases. Moreover, the urban environment of people who live in low-SES neighborhoods, as it relates to food, is much different from that in affluent areas. Not only are fast-food outlets and convenience stores commonplace in disadvantaged neighborhoods [11,153-155], but outdoor advertising of high-energy, low-nutrient foods and beverages is more prevalent there [156-158]. Not surprisingly, because there is little dispute that well-financed, targeted marketing is effective [159], brand name logo recognition of major fast-food outlets is high among children in low-SES neighborhoods [160].

Research also shows, on the one hand, that people in a lowered mood state are less likely to focus on the future health consequences of dietary decisions. Positive mood, on the other hand, increases the likelihood that an individual will not discount the future implications of what they are eating in the here and now [161]. This type of research, under the umbrella term of *delayed* (or *temporal*) *discounting*, is important in the context of the urban nutritional environment. Humans are prone to discount the value of future rewards and prioritize smaller but more immediate rewards. The strength of discounting, however, is associated with impulsivity, depression, obesity and unhealthy lifestyle habits [153,162-165]. Minimal discounting of future considerations (that is, placing a high value on the future) is associated with a healthier diet and higher physical activity levels [166]. The overall cognitive load within an urban environment may magnify delayed discounting [167]. Subtle aspects of the urban environment, such as subconscious exposure to fast-food logos or even answering surveys in the general proximity of an urban fast-food outlet (vs full-service restaurant), increase impatience and encourage the discounting of future rewards for immediate gain. The immediate gain in this case might be the pleasure associated with low-nutrient, highly palatable foods and beverages. Moreover, individuals who reside in neighborhoods with high concentrations of fast-food outlets are more likely to prefer smaller immediate rewards over larger future gains [155].

Moving along this continuum, emerging research shows that food advertising as a driver of unhealthy food consumption is more pronounced when cognitive load is high (that is, distracting mental demands) and that those with lower SES backgrounds are more susceptible to the effects of advertising under cognitive load [168]. New research also confirms that excessive fast-food consumption is indeed attributable to the neighborhood food environment, with as much as 31% of the variance in excessive consumption attributable to living in urban areas with a moderate or high density of fast-food outlets [169]. However, fruit and vegetable consumption is markedly diminished in neighborhoods with a high clustering of fast-food outlets [170,171]. Surrogate physiological markers of fruit and vegetable intake (for example, serum carotenoids) support the notion that adults who reside in deprived or lower SES neighborhoods are less likely to consume these foods or may displace them with unhealthy choices [172,173].

Connected to the marketing forces influencing a pull away from traditional dietary habits is screen-based media consumption. Excess screen-based media consumption and so-called technostress have recently been linked to poor psychological health [174-182], and research shows that in neighborhoods where walkability is less than optimal, screen time is higher [183,184]. Moreover, children who reside in urban environments [185] and deprived neighborhoods [186] have higher daily screen time than their rural or affluent counterparts. Higher screen time and depression could merely be a matter of association with less physical activity [187]; however, research shows that screen time (independently of physical activity) is significantly associated with the consumption of high-energy, low-nutrient foods and beverages [188]. Prospective research shows that baseline screen time predicts higher consumption of sugar-rich beverages when queried 20 to 24 months later [189,190]. Similar results have been reported concerning baseline screen time and subsequent dietary habits in high school students and young adults: More screen time was found to predict consumption of fast-food, snacks and high-energy foods and beverages 5 years later [191]. The ways in which screen media can influence nutritional imbalances by encouraging high-energy, low-nutrient food consumption—via distraction of the activity itself and marketing of such foods during the activity [192-195]—is an area of research which requires expansion.

Evening screen media consumption, and even low levels of light at night (LAN), may suppress melatonin levels and compromise sleep [196]. Compromised sleep, too, has not only been linked to depression but also is associated with a prioritization of unhealthy foods choices [197,198], and, at least in experimental models, LAN results in a more pronounced inflammatory response to high-fat foods [199]. Indeed, circadian rhythm disruption appears to be a distinct biological stressor that interacts with Westernized dietary patterns in the promotion of intestinal permeability and the loss of normal intestinal microbiota [200,201].

Despite the research indicating that the consumption of energy-dense, high-fat, high-sugar and sodium-rich foods may be a highly palatable form of self-medication, the results of collection of dietary records and mood scores over a weekly period suggest that (in healthy university students) consumption of such foods increases negative mood 48 hours after consumption [202]. Additionally, food and beverage consumption is often a social activity. A greater understanding of the ways in which social isolation can minimize dietary diversity [203] or social support can facilitate healthy dietary patterns [204] is an essential area of research in nutritional psychiatry.

As a segue to our discussion regarding the evolutionary mismatch and nutritional anthropology, it is important to point out the cultural aspects of traditional dietary patterns as exemplified by the Mediterranean and Japanese models. It is not by chance that the Japanese and Mediterranean diets are each officially listed by the United Nations Educational, Scientific and Cultural Organization (UNESCO) on its Lists of Intangible Cultural Heritage in Need of Urgent Safeguarding and Register of Best Safeguarding Practices [205]. Both of these dietary patterns are steeped in tradition—nutritionally and otherwise. The listing by UNESCO specifically makes reference to the shared family and community experience in the consumption of these respective diets, including the ways in which dietary practices have been passed down from generation to generation. In other words, the cultural aspects of the diets are integral to adherence.

Cultural influences remain a driver of traditional dietary patterns; however, the Westernization of Japanese and Mediterranean diets appears to be corroding the culture of dietary tradition in both regions [206-208]. Lower rates of adherence to the Mediterranean diet have been reported to be common among urban youth in Italy and Greece compared to their rural counterparts [209,210]. Lower amounts of screen time are associated with higher adherence to the Mediterranean diet in Italian children [211]. The extent to which individual interest in traditional dietary patterns is associated with both social capital and psychological distress is a subject of ongoing research [212].

The relationships between healthy dietary habits and mental health are obviously complex. They weave their way through many aspects of modern technology and culture. The extent to which these nutritionally related environmental challenges ultimately contribute to the causation of mental disorders or present a barrier to effective treatment is a question in clear need of exploration. We hope that this will be the legacy of the field of nutritional psychiatry. In the meantime, it seems safe to say that the environmental challenges discussed above, those that might accelerate the adoption of Western dietary patterns, are colliding with an ancestral past that is still dictating human physiology [213].

### Evolutionary mismatch?

Nutritional anthropology has taught us much concerning the makeup of ancestral dietary patterns. Undoubtedly, the ancestral diets were regionally varied, and it seems clear that there was no archetypal “Stone Age cuisine.” However, despite their differences, ancestral diets largely included a high intake of plant foods (rich in fiber and phytochemicals and facilitating a net base production), animal foods and a diverse range of commensal bacteria. Ancestral diets were also united by their absence of highly processed foods containing high sodium, added fats and refined sugar. In other words, the anthropological evidence concerning ancestral diets indicates that their regional and seasonal differences were minor compared to the ways in which they differ from the modern nutritional landscape [214,215]. With this in mind, we can move toward discussions of evolutionary perspectives and epigenetic opportunities. After some background and historical framing, we next focus on three components of ancestral and traditional dietary practices: cooking techniques, dietary acid load and microbiota.

Evolutionary explanations for contemporary increases in psychopathologies include the mismatch theory. Broadly, this proposal suggests that there are gaps between the physiological and psychological requirements of individuals, as determined through millennia of adaptation to natural and social environments, and the ability of the modern environment to help fulfill those brain or mental health–related needs [29,216]. The likelihood of mismatch is increased, according to theory, when culture and technology change more rapidly than human biological evolution [217]. Some of the lifestyle modifications associated with rapid technological and cultural changes—diminished physical activity, daily hassles and low-grade psychological stress, increased consumption of low-nutrient and energy-dense foods, loss of biodiversity in microbial contact—could chronically and unnecessarily stoke an immune defense system that is stuck in its ancestral past [218]. The ancestral immune system remains biologically well-adapted to preventing pathogen invasion, limiting acute infections, working with commensal microbes and assisting in programming the development of the nervous system [219]. However, its ability to perform these and other functions, as it has done for millennia, may now involve collateral damage in the form of allergies, asthma, autoimmune conditions and increased risk of neuropsychiatric disorders [220-222].

The notion that modern dietary intake is at odds with our ancestral past, and that this disconnect has health-related consequences, is not new. In the early part of the 20th century, a small minority of medical writers were sounding alarms concerning the implications of a shift away from traditional diets. In 1922, the editors of the *Medical Standard* journal issued a stern warning concerning the increased consumption of the “output of the candy shop, soda fountain or pastry shop,” or, as they called it, “denatured, devitalized and demoralized foodstuffs.” More specifically, the editors’ argument was that such foods and beverages were at odds with optimal physiological functioning: “In other words, any kind of food is unfit to eat when its composition, through some artificial form of kitchen treatment, has become hostile to physiological life.… What is not for me is against me.… Never before in the world’s history of individual evolution were so many temptations put before him in the field of appetite and sensuous appeal as today” [223]. Also at this time, some experts were examining the dietary practices of people maintaining an isolated or indigenous existence [224], and others, such as noted nutritional scientist Frances Stern (1873–1947; later she would have a clinic at Tufts Medical Center (Boston, MA, USA) dedicated in her name), were assisting disadvantaged immigrants with ways in which to maintain their traditional dietary habits in an affordable way [225].

Stern, for her part, addressed the leaders of North American psychiatry at an annual conference in 1929, “hoping that the leaders there may guide the nutritionist to greater wisdom in the treatment of her patient.… In the field of dietotherapy, however, there is need of further light from psychiatry with regard to the interpretation of the mental life of patients, and the nutritionist is looking to the mental hygiene movement for research into the nature of the relationships between food and disturbances of the emotional life” [226]. Only today, the better part of a century later, has a response to Stern’s plea begun to take shape. From the perspective of nutritional anthropology, it was not until the 1980s that a scientific framework began to emerge, one in which shifts away from traditional dietary habits were at the core of the mismatch discussions and connections to chronic degenerative diseases [227].

Dietary patterns are more than simply reflections of macronutrients. In addition to the cultural aspects alluded to earlier, they involve methods of preparation. Experts in anthropology have noted that cooking techniques involving water—steaming and boiling—were widespread in our ancestral past. Indeed, although the precise dating of so-called Stone Age soups and stews remains unknown, they appear to be a minimum of 20,000 years old [228]. Some experts suggest that the use of steam to release necessary fat from animal bones may have been in practice 50,000 years ago or more [229,230]. Suffice it to say that water-based cooking is an ancient practice. Those investigating the marked shift away from traditional dietary patterns in China and elsewhere have noted that the shifts toward processed foods and less potassium-rich foods are associated with changes in cooking technique. Specifically, there has been a decline in the consumption of foods prepared by steaming or boiling [4]. The traditional Japanese diet, one currently undergoing erosion, has included steamed and boiled foods since its early origins [231]. Indeed, the *dashi* (soup stock) is said to be the backbone of Japanese cuisine. *Dashi* made with flakes of dried bonito has been shown to improve mental outlook in randomized, controlled human trials [232] and to reduce anxiety-like behavior in experimental rodent studies [233].

One of the consequences of a shift away from water-based cooking to that of high heat in the absence of water (that is, baking, roasting, grilling, frying) is that it encourages the formation of advanced glycation end products (AGEs). Not to be confused with heterocyclic amines (HCAs) and polycyclic aromatic hydrocarbons (PAHs) produced by grilling meats (HCAs and PAHs experimentally linked to cancer), AGEs are highly oxidant compounds formed through the nonenzymatic reaction between reducing sugars and free amino acids. AGEs form in the body under normal metabolic circumstances; however, oxidative stress and inflammation are a well-known consequence of AGE levels rising beyond normal limits. The results of recent investigations have shown that the preformed AGEs in foods also add to the endogenous AGE burden. When considering the Westernized dietary pattern, with its reliance on foods thermally processed with high (dry) heat to enhance flavor, appearance and color, it should not be surprising that many of its foods are very high in AGEs [234]. The results of human studies have shown that a shift toward stewing, steaming, poaching and boiling of foods (lowering the AGE burden by approximately 50%) significantly reduces systemic inflammation and oxidative stress [235,236]. Most of the research has been focused on type 2 diabetes and obesity, with improvements in insulin sensitivity noted in both populations after a switch to a low-AGE diet [237,238]. Interestingly, people with depression and schizophrenia have low blood levels of soluble receptor for AGEs, a receptor tasked with binding and essentially halting some of the destructive consequences of AGEs [239,240]. The emerging research related to AGEs forces us to look at not only dietary patterns but also the ways in which the foods within these patterns are prepared.

Another understudied aspect of the relevance of ancestral diets in contemporary mental health relates to the dietary acid load. Acid–base homeostasis is an example of a tightly controlled set of physiological mechanisms, checks and balances that are almost certainly a by-product of the Paleolithic nutritional environment [241]. The best available evidence suggests that the diets of our early East African ancestors were predominantly net base-producing [242]. The Westernized diet, with its heavy protein (amino acid) load, sodium intake and relative absence of fruits and vegetables (typically rich in buffering potassium, bicarbonate precursors, magnesium and calcium), has been linked to what has been described as a low-grade systemic metabolic acidosis in otherwise healthy adults [243]. Notwithstanding the pseudoscientific extrapolation of the low-grade acidosis proposal into so-called alkaline diets by lay writers, intriguing prospective research has shown that dietary acid load increases subsequent risk of type 2 diabetes [244]. Acid load has also been linked to cardiovascular health [245], elevated body weight, increased waist circumference and a lower percentage of lean body mass [246-248].

In our current context of mental health, it is noteworthy that a diet rich in potassium (compared to the DASH diet (Dietary Approaches to Stop Hypertension) and a separate group in a high-calcium diet) was found to improve depression, tension, energy and the Profile of Mood States global score in otherwise healthy adults over the course of 1 month [249]. Also relevant to mental health is a specific physiological change induced by a high-acid load, fast-food type diet. In otherwise healthy adults who consumed an acidic, Western-type diet for 9 days, researchers neutralized the diet with bicarbonate supplements (while subjects maintained the high-acid-load diet) and found that cortisol levels were reduced significantly vs controls maintaining the diet [250]. This result suggests that the high acid load itself is a stressor because cortisol elevation can contribute to inflammation, oxidative stress and lowered mood states [251].

Because the acid–base balance in human blood and other tissue is so tightly regulated, with the exceptions of kidney disease and acute, overt metabolic acidosis, it would be tempting to dismiss the relevancy of dietary factors in regard to their ability to influence pH in and around tissue. However, despite unaltered blood pH, the chronic administration of oral bicarbonate has been shown to increase the extracellular pH of tumors, subsequently reducing the *in vivo* number and size of tumor metastases. Ultimately in those studies, the reductions in metastases after oral bicarbonate led to increased survival rates of the animals with tumors [252,253]. In the general population, dietary acid load is associated with lower serum bicarbonate levels [254]. Returning to mental health, it has been shown that the ability of inhaled carbon dioxide to induce fear can be diminished by systemically administered bicarbonate. The amygdala is a highly sensitive chemosensor and contains within it the acid-sensing ion channel 1a (ASIC1a). Direct reductions of pH within the amygdala can evoke fear behavior, whereas inhibition of the ASIC1a receptor has been shown to have antidepressant-like effects in various stress models [255,256].

Could there be connections between behavior, dietary acid load (and the low end of the normal physiological blood pH range from 7.36 to 7.38), ASIC1a sensitivity and the low serum bicarbonate levels associated with dietary acid load? Perhaps. In the meantime, we do know that potassium-rich diets are less frequently consumed by people with depression [257] and those in lower SES groups [258]. This is an area ripe for research. Related to this topic is the emerging research showing that even mild states of dehydration can negatively influence mood and cognition [259].

An aspect of evolutionary medicine that has ignited a spark in the international research community is a possible connection between the microbiome and mental health. This area of research, at least in its contemporary tone, has many of its original roots in the 1980s hygiene hypothesis, that which suggested that the global rise in allergic disease could be related to diminished opportunity for early-life exposure to pathogenic microbe exposure via increased hygiene and use of antibiotics as well as smaller family size [260]. A decade later, after a landmark 1998 paper by Swedish physician Agnes Wold, the focus shifted toward a more general gut microbial deprivation hypothesis, one that included “low exposure to bacteria *via* food or the environment in general. All this results in an ‘abnormally’ stable microflora” in Westernized nations [261]. The traditional focus on harmful microbes shifted toward lactic acid bacteria and commensals, with novel scientific frameworks bridging the immune system to emotional health via nonpathogenic microbes [262,263].

The shift toward an abnormally stable (inflammatory) gut microbiota in Westernized nations became more evident when sophisticated 16S rRNA sequencing techniques allowed detailed stool analyses to be conducted. The results of recent studies have consistently shown that, compared to Westernized urbanites, rural dwellers and hunter-gatherers in Africa and South America have higher levels of microbial richness and diversity. The available evidence suggests that diet, rather than hygiene *per se*, is the key component to microbial diversity [264-267]. The results of analysis of the microbiome within ancient human coprolite (paleostool) samples indicate more alignment with the modern hunter-gatherer than with the urban dweller in a developed nation [268]. Because ancestral diets seem to set up a gut microbial diversity that is distinct from that currently being associated with obesity and type 2 diabetes [269], we once again arrive at a list of further questions pertaining to the ways in which traditional diets influence mental health.

Scientific interest in lifestyle medicine in general, and nutritional influences related to DOHaD in particular, has grown rapidly in the past several years [270,271]. These discussions have often focused on obesity, cardiovascular disease, type 2 diabetes and allergic diseases. Yet, there are still only limited discussions related to parental and early-life nutrition, epigenetic changes, microevolution, life-course and even transgenerational mental health [272-274]. Children born preterm (vs peers delivered at full term) are at significantly higher risk for developing mental disorders [275]; therefore, studies showing that healthy diets, at the time of conception [276] or during pregnancy [277], are associated with reduced risk of preterm delivery may have enormous implications for mental health. Although allergy is most often the first NCD to present itself in the clinical realm, subtle and even not-so-subtle behavioral alterations may predict subsequent allergic disease risk [278,279].

It is not our desire to privilege mental health in the DOHaD discussions; rather, we wish to highlight the notion that a broader view is required, one that avoids silos and encourages collaboration. It seems very likely that the same evolutionary mismatch implicated in the global rise in allergic and autoimmune diseases is not distinct from that which seems to be at play in mental disorders [280]. Early-life immune programming via diet and microbes appears to be a universal application related to NCDs. The recent evidence showing that unhealthy maternal dietary choices predict subsequent childhood behavioral problems [70] is both groundbreaking and a source of optimism. It indicates that, despite the environmental challenges discussed earlier, things can be fixed. Indeed, there is an increasing sense of optimism regarding the opportunities for the prevention of mental disorders globally [281,282].

## Summary

Over the past several years, there has been a rapid growth in high-quality research related to nutrition and mental health. An area that has suffered neglect is finally starting to take shape, albeit in a preliminary fashion. Given the global changes described above—urbanicity, climate change and the globalization of the food industry resulting in profound shifts from traditional dietary patterns—there is a sense of urgency to bringing more efficiency and strength to the process of determining the ways in which overall dietary patterns, specific nutritional elements and/or multinutrient interventions can influence mental health.

Compromised mental health in both the broad sense (quality of life) and the more narrow clinical sense (disorders diagnosable based on the definitions published in the *Diagnostic and Statistical Manual of Mental Disorders, Fifth Edition*) is highly comorbid with NCDs, and poor physical health is a particularly strong predictor of poor mental health [283]. Moreover, many of the high-prevalence NCDs share a multitude of underlying pathophysiological pathways with mental illnesses, including immune dysfunction and oxidative stress [59]. One need only pick a medical specialty, from cardiology and dermatology to gastroenterology and rheumatology, and it is plain to see how mental health presents itself as a variable of importance. However, nutrition is also emerging as a variable that uniquely interfaces with both mental health and some of the more notable conditions within these disciplines. Put another way, emerging research suggests that nutrition not only matters directly with regard to the conditions treated within various medical disciplines but also has the potential to influence mental outlook and mental disorders (improving or compromising, depending on intake) that can otherwise contribute to the ultimate outcomes of what are viewed as physical illnesses [284]. We cannot ignore this, particularly as it is becoming increasingly clear that diminished mental outlook and elevated perceptions of stress are drivers of unhealthy eating habits [285-287].

It is not the purpose of our discussion here concerning the interface between nutritional psychiatry and physiological anthropology to systematically review the outcomes and/or potential pathophysiological pathways in minute detail, nor is it to provide a critical assessment of the state of the nutritional psychiatry science in the context of current evidence-based mental health interventions. Our primary message is that the field of nutritional psychiatry is rapidly developing and that, although for the moment it primarily involves global researchers in the fields of nutrition, mental health, population health and epidemiology, this list will surely expand.

Emerging groups such as the International Society for Nutritional Psychiatry Research aim to foster awareness, collaboration and ultimately meaningful clinical translation. Nutrition does not stand alone; it is intimately woven throughout social, cultural, economic, technological, behavioral and other (ancestral past vs present) environmental fabrics with which physiological anthropology concerns itself. As we have tried to make clear in this review, nutritional psychiatry is not a psychiatric specialty *per se*; on the contrary, the emerging research on the topic has been driven by an eclectic group of specialists in a wide variety of disciplines.

Time is no longer on the side of researchers. Global urbanization and an impending mental health crisis are a tandem juggernaut moving at rapid speed. Its destructive force will not be slowed down by additional silo-style research. Although growing in quality and providing cause for optimism, the current research remains scattered in various disciplines and lacks the collective push that is required in the process of translational medicine. Research studies published in isolation, no matter how elegant, ultimately make the job of translation and policy-making more difficult. We can surely conclude that things do not get better, in the scientific sense, by further neglect and disorganization of approach.

## Competing interests

ACL has received consulting fees from Genuine Health Inc (Toronto, ON, Canada). FJN has received grant and research support from the Brain & Behaviour Research Institute, the National Health and Medical Research Council, Australian Rotary Health, the Geelong Medical Research Foundation, the Ian Potter Foundation, Eli Lilly, the Meat and Livestock Board Australia and The University of Melbourne, and has been a paid speaker for Sanofi-Synthelabo, Janssen Cilag, Servier, Pfizer, Health Ed, Network Nutrition, Angelini Farmaceutica and Eli Lilly.

## Authors' contributions

Both authors made significant contributions to the manuscript. ACL provided the primary framework for the content. FNJ provided oversight and important intellectual content within each major section. Both authors read and approved the final manuscript.

## References

[B1] Seto KC Güneralp B Hutyra LR Global forecasts of urban expansion to 2030 and direct impacts on biodiversity and carbon pools Proc Natl Acad Sci U S A 2012 109 16083 16088 22988086 10.1073/pnas.1211658109 PMC3479537

[B2] Nations U World Urbanization Prospects (2011 revision) 2012 New York: Author

[B3] Cyril S Oldroyd JC Renzaho A Urbanisation, urbanicity, and health: a systematic review of the reliability and validity of urbanicity scales BMC Public Health 2013 13 513 23714282 10.1186/1471-2458-13-513 PMC3671972

[B4] Zhai FY Du SF Wang ZH Zhang JG Du WW Popkin BM Dynamics of the Chinese diet and the role of urbanicity, 1991–2011 Obes Rev 2014 15 Suppl 1 16 26 24341755 10.1111/obr.12124 PMC3868998

[B5] Levine JA McCrady SK Boyne S Smith J Cargill K Forrester T Non-exercise physical activity in agricultural and urban people Urban Stud 2011 48 2417 2427 22073428 10.1177/0042098010379273

[B6] Delisle H Ntandou-Bouzitou G Agueh V Sodjinou R Fayomi B Urbanisation, nutrition transition and cardiometabolic risk: the Benin study Br J Nutr 2012 107 1534 1544 22115429 10.1017/S0007114511004661

[B7] Ng SW Howard AG Wang HJ Su C Zhang B The physical activity transition among adults in China: 1991–2011 Obes Rev 2014 15 Suppl 1 27 36 24341756 10.1111/obr.12127 PMC3869092

[B8] Bedrosian TA Nelson RJ Influence of the modern light environment on mood Mol Psychiatry 2013 18 751 757 23711982 10.1038/mp.2013.70

[B9] Popkin BM Contemporary nutritional transition: determinants of diet and its impact on body composition Proc Nutr Soc 2011 70 82 91 21092363 10.1017/S0029665110003903 PMC3029493

[B10] Popkin BM Adair LS Ng SW Global nutrition transition and the pandemic of obesity in developing countries Nutr Rev 2012 70 3 21 22221213 10.1111/j.1753-4887.2011.00456.x PMC3257829

[B11] Richardson AS Boone-Heinonen J Popkin BM Gordon-Larsen P Are neighbourhood food resources distributed inequitably by income and race in the USA? Epidemiological findings across the urban spectrum. BMJ Open 2012 2 e000698 10.1136/bmjopen-2011-000698 PMC3329604 22505308

[B12] Whiteford HA Degenhardt L Rehm J Baxter AJ Ferrari AJ Erskine HE Charlson FJ Norman RE Flaxman AD Johns N Burstein R Murray CJ Vos T Global burden of disease attributable to mental and substance use disorders: findings from the Global Burden of Disease Study 2010 Lancet 2013 382 1575 1586 23993280 10.1016/S0140-6736(13)61611-6

[B13] Baxter AJ Patton G Scott KM Degenhardt L Whiteford HA Global epidemiology of mental disorders: What are we missing? PLoS One 2013 8 e65514 23826081 10.1371/journal.pone.0065514 PMC3691161

[B14] Murray CJ Vos T Lozano R Naghavi M Flaxman AD Michaud C Ezzati M Shibuya K Salomon JA Abdalla S Aboyans V Abraham J Ackerman I Aggarwal R Ahn SY Ali MK Alvarado M Anderson HR Anderson LM Andrews KG Atkinson C Baddour LM Bahalim AN Barker-Collo S Barrero LH Bartels DH Basáñez MG Baxter A Bell ML Benjamin EJ Disability-adjusted life years (DALYs) for 291 diseases and injuries in 21 regions, 1990–2010: a systematic analysis for the Global Burden of Disease Study 2010 Lancet 2012 380 2197 2223 23245608 10.1016/S0140-6736(12)61689-4

[B15] Jacobi F Höfler M Siegert J Mack S Gerschler A Scholl L Busch MA Hapke U Maske U Seiffert I Gaebel W Maier W Wagner M Zielasek J Wittchen HU Twelve-month prevalence, comorbidity and correlates of mental disorders in Germany: the Mental Health Module of the German Health Interview and Examination Survey for Adults (DEGS1-MH) Int J Methods Psychiatr Resin press. doi:10.1002/mpr.1439 10.1002/mpr.1439 PMC6878234 24729411

[B16] Lederbogen F Kirsch P Haddad L Streit F Tost H Schuch P Wüst S Pruessner JC Rietschel M Deuschle M Meyer-Lindenberg A City living and urban upbringing affect neural social stress processing in humans Nature 2011 474 498 501 21697947 10.1038/nature10190

[B17] Penkallas AM Kohler S Urbanicity and mental health in Europe: a systematic review Eur J Mental Healthin press

[B18] Yiengprugsawan V Caldwell BK Lim LL Seubsman S Sleigh AC Lifecourse urbanization, social demography, and health outcomes among a national cohort of 71,516 adults in Thailand Int J Popul Res 2011 2011 464275 22428087 10.1155/2011/464275 PMC3303129

[B19] Prina AM Ferri CP Guerra M Brayne C Prince M Prevalence of anxiety and its correlates among older adults in Latin America, India and China: cross-cultural study Br J Psychiatry 2011 99 485 491 22016438 10.1192/bjp.bp.110.083915 PMC3227807

[B20] Prina AM Ferri CP Guerra M Brayne C Prince M Co-occurrence of anxiety and depression amongst older adults in low- and middle-income countries: findings from the 10/66 study Psychol Med 2011 41 2047 2056 21466747 10.1017/S0033291711000444

[B21] Trivedi JK Sareen H Dhyani M Rapid urbanization - its impact on mental health: a south Asian perspective Indian J Psychiatry 2008 50 161 165 19742238 10.4103/0019-5545.43623 PMC2738359

[B22] Jacka FN Sacks G Berk M Allender S Food policies for physical and mental health BMC Psychiatry 2014 14 132 24884515 10.1186/1471-244X-14-132 PMC4026829

[B23] Redshaw CH Stahl-Timmins WM Fleming LE Davidson I Depledge MH Potential changes in disease patterns and pharmaceutical use in response to climate change J Toxicol Environ Health B Crit Rev 2013 16 285 320 23909463 10.1080/10937404.2013.802265 PMC3756629

[B24] Dewing S Tomlinson M le Roux IM Chopra M Tsai AC Food insecurity and its association with co-occurring postnatal depression, hazardous drinking, and suicidality among women in peri-urban South Africa J Affect Disord 2013 150 460 465 23707034 10.1016/j.jad.2013.04.040 PMC3762324

[B25] Melchior M Chastang JF Falissard B Galéra C Tremblay RE Côté SM Boivin M Food insecurity and children’s mental health: a prospective birth cohort study PLoS One 2012 7 e52615 23300723 10.1371/journal.pone.0052615 PMC3530436

[B26] Bloom SR Kuhajda FP Laher I Pi-Sunyer X Ronnett GV Tan TM Weigle DS The obesity epidemic: pharmacological challenges Mol Interv 2008 8 82 98 18403653 10.1124/mi.8.2.6

[B27] Hidaka BH Depression as a disease of modernity: explanations for increasing prevalence J Affect Disord 2012 140 205 214 22244375 10.1016/j.jad.2011.12.036 PMC3330161

[B28] Colla J Buka S Harrington D Murphy JM Depression and modernization: a cross-cultural study of women Soc Psychiatry Psychiatr Epidemiol 2006 41 271 279 16520885 10.1007/s00127-006-0032-8

[B29] Logan AC Selhub EM *Vis Medicatrix naturae*: does nature “minister to the mind”? Biopsychosoc Med 2012 6 11 22472137 10.1186/1751-0759-6-11 PMC3353853

[B30] Streit F Haddad L Paul T Frank J Schäfer A Nikitopoulos J Akdeniz C Lederbogen F Treutlein J Witt S Meyer-Lindenberg A Rietschel M Kirsch P Wüst S A functional variant in the neuropeptide S receptor 1 gene moderates the influence of urban upbringing on stress processing in the amygdala Stress 2014 17 352 361 24800784 10.3109/10253890.2014.921903

[B31] Peen J Schoevers RA Beekman AT Dekker J The current status of urban–rural differences in psychiatric disorders Acta Psychiatr Scand 2010 121 84 93 19624573 10.1111/j.1600-0447.2009.01438.x

[B32] Rudolph KE Stuart EA Glass TA Merikangas KR Neighborhood disadvantage in context: the influence of urbanicity on the association between neighborhood disadvantage and adolescent emotional disorders Soc Psychiatry Psychiatr Epidemiol 2014 49 467 475 23754682 10.1007/s00127-013-0725-8 PMC6438365

[B33] Bijl RV de Graaf R Hiripi E Kessler RC Kohn R Offord DR Ustun TB Vicente B Vollebergh WA Walters EE Wittchen HU The prevalence of treated and untreated mental disorders in five countries Health Aff 2003 22 122 133 10.1377/hlthaff.22.3.122 12757277

[B34] Yang G Wang Y Zeng Y Gao GF Liang X Zhou M Wan X Yu S Jiang Y Naghavi M Vos T Wang H Lopez AD Murray CJ Rapid health transition in China, 1990–2010: findings from the Global Burden of Disease Study 2010 Lancet 2013 381 1987 2015 23746901 10.1016/S0140-6736(13)61097-1 PMC7159289

[B35] Lee KS Park JH Burden of disease in Korea during 2000–10 J Public Health 2014 36 225 234 10.1093/pubmed/fdt056 23759601

[B36] Mokdad AH Jaber S Aziz MI AlBuhairan F AlGhaithi A AlHamad NM Al-Hooti SN Al-Jasari A AlMazroa MA AlQasmi AM Alsowaidi S Asad M Atkinson C Badawi A Bakfalouni T Barkia A Biryukov S El Bcheraoui C Daoud F Forouzanfar MH Gonzalez-Medina D Hamadeh RR Hsairi M Hussein SS Karam N Khalifa SE Khoja TA Lami F Leach-Kemon K Memish ZA The state of health in the Arab world, 1990–2010: an analysis of the burden of diseases, injuries, and risk factors Lancet 2014 383 309 320 24452042 10.1016/S0140-6736(13)62189-3

[B37] Sartorius N Cimino L The co-occurrence of diabetes and depression: an example of the worldwide epidemic of comorbidity of mental and physical illness Ann Acad Med Singapore 2012 41 430 431 23138138

[B38] Lin CH Lee YY Liu CC Chen HF Ko MC Li CY Urbanization and prevalence of depression in diabetes Public Health 2012 126 104 111 22178148 10.1016/j.puhe.2011.10.006

[B39] van Reedt Dortland AK Giltay EJ van Veen T Zitman FG Penninx BW Longitudinal relationship of depressive and anxiety symptoms with dyslipidemia and abdominal obesity Psychosom Med 2013 75 83 89 23197842 10.1097/PSY.0b013e318274d30f

[B40] Thurston RC Rewak M Kubzansky LD An anxious heart: anxiety and the onset of cardiovascular diseases Prog Cardiovasc Dis 2013 55 524 537 23621962 10.1016/j.pcad.2013.03.007

[B41] Nielsen TJ Vestergaard M Christensen B Christensen KS Larsen KK Mental health status and risk of new cardiovascular events or death in patients with myocardial infarction: a population-based cohort study BMJ Open 2013 3 e003045 10.1136/bmjopen-2013-003045 PMC3733312 23913773

[B42] Balázs J Miklósi M Keresztény A Hoven CW Carli V Wasserman C Apter A Bobes J Brunner R Cosman D Cotter P Haring C Iosue M Kaess M Kahn JP Keeley H Marusic D Postuvan V Resch F Saiz PA Sisask M Snir A Tubiana A Varnik A Sarchiapone M Wasserman D Adolescent subthreshold-depression and anxiety: psychopathology, functional impairment and increased suicide risk J Child Psychol Psychiatry 2013 54 670 677 23330982 10.1111/jcpp.12016

[B43] Berk M Sarris J Coulson CE Jacka FN Lifestyle management of unipolar depression Acta Psychiatr Scand Suppl 2013 443 38 54 23586875 10.1111/acps.12124

[B44] Penn E Tracy DK The drugs don’t work? Antidepressants and the current and future pharmacological management of depression Ther Adv Psychopharmacol 2012 2 179 188 23983973 10.1177/2045125312445469 PMC3736946

[B45] Khan A Faucett J Lichtenberg P Kirsch I Brown WA A systematic review of comparative efficacy of treatments and controls for depression PLoS One 2012 7 e41778 22860015 10.1371/journal.pone.0041778 PMC3408478

[B46] Spielmans GI Kirsch I Drug approval and drug effectiveness Annu Rev Clin Psychol 2014 10 741 766 24329178 10.1146/annurev-clinpsy-050212-185533

[B47] Cuijpers P Smit F Bohlmeijer E Hollon SD Andersson G Efficacy of cognitive-behavioural therapy and other psychological treatments for adult depression: meta-analytic study of publication bias Br J Psychiatry 2010 196 173 178 20194536 10.1192/bjp.bp.109.066001

[B48] Cuijpers P Berking M Andersson G Quigley L Kleiboer A Dobson KS A meta-analysis of cognitive-behavioural therapy for adult depression, alone and in comparison with other treatments Can J Psychiatry 2013 58 376 385 23870719 10.1177/070674371305800702

[B49] Furukawa TA Noma H Caldwell DM Honyashiki M Shinohara K Imai H Chen P Hunot V Churchill R Waiting list may be a nocebo condition in psychotherapy trials: a contribution from network meta-analysis Acta Psychiatr Scandin press. doi:10.1111/acps.12275 10.1111/acps.12275 24697518

[B50] Cuijpers P Turner EH Mohr DC Hofmann SG Andersson G Berking M Coyne J Comparison of psychotherapies for adult depression to pill placebo control groups: a meta-analysis Psychol Med 2014 44 685 695 23552610 10.1017/S0033291713000457

[B51] Thomas L Kessler D Campbell J Morrison J Peters TJ Williams C Lewis G Wiles N Prevalence of treatment-resistant depression in primary care: cross-sectional data Br J Gen Pract 2013 63 e852 e858 24351501 10.3399/bjgp13X675430 PMC3839394

[B52] Jacka FN Reavley NJ Prevention of mental disorders: evidence, challenges and opportunities BMC Med 2014 12 75 24886356 10.1186/1741-7015-12-75 PMC4014629

[B53] Jacka FN Cherbuin N Anstey KJ Butterworth P Dietary patterns and depressive symptoms over time: examining the relationships with socioeconomic position, health behaviours and cardiovascular risk PLoS One 2014 9 e87657 24489946 10.1371/journal.pone.0087657 PMC3906192

[B54] Gesch B Adolescence: Does good nutrition = good behavior? Nutr Health 2014 22 55 65 24500658 10.1177/0260106013519552 PMC4817227

[B55] Sihvola N Korpela R Henelius A Holm A Huotilainen M Müller K Poussa T Pettersson K Turpeinen A Peuhkuri K Breakfast high in whey protein or carbohydrates improves coping with workload in healthy subjects Br J Nutr 2013 110 1712 1721 23591085 10.1017/S0007114513000779

[B56] Belcaro G Luzzi R Dugall M Ippolito E Saggino A Pycnogenol® improves cognitive function, attention, mental performance and specific professional skills in healthy professionals age 35–55 J Neurosurg Sciin press 24675223

[B57] Sarris J O’Neil A Coulson CE Schweitzer I Berk M Lifestyle medicine for depression BMC Psychiatry 2014 14 107 24721040 10.1186/1471-244X-14-107 PMC3998225

[B58] Moylan S Berk M Dean OM Samuni Y Williams LJ O’Neil A Hayley AC Pasco JA Anderson G Jacka FN Maes M Oxidative & nitrosative stress in depression: Why so much stress? Neurosci Biobehav Rev 2014 45C 46 62 24858007 10.1016/j.neubiorev.2014.05.007

[B59] Berk M Williams LJ Jacka FN O’Neil A Pasco JA Moylan S Allen NB Stuart AL Hayley AC Byrne ML Maes M So depression is an inflammatory disease, but where does the inflammation come from? BMC Med 2013 11 200 24228900 10.1186/1741-7015-11-200 PMC3846682

[B60] Sizonenko SV Babiloni C Sijben JW Walhovd KB Brain imaging and human nutrition: which measures to use in intervention studies? Adv Nutr 2013 4 554 556 24038255 10.3945/an.113.004283 PMC3771147

[B61] Jacka FN Mykletun A Berk M Bjelland I Tell GS The association between habitual diet quality and the common mental disorders in community-dwelling adults: the Hordaland Health Study Psychosom Med 2011 73 483 490 21715296 10.1097/PSY.0b013e318222831a

[B62] Jacka FN Pasco JA Mykletun A Williams LJ Hodge AM O’Reilly SL Nicholson GC Kotowicz MA Berk M Association of western and traditional diets with depression and anxiety in women Am J Psychiatry 2010 167 305 311 20048020 10.1176/appi.ajp.2009.09060881

[B63] Sánchez-Villegas A Delgado-Rodríguez M Alonso A Schlatter J Lahortiga F Serra Majem L Martínez-González MA Association of the Mediterranean dietary pattern with the incidence of depression: the Seguimiento Universidad de Navarra/University of Navarra follow-up (SUN) cohort Arch Gen Psychiatry 2009 66 1090 1098 19805699 10.1001/archgenpsychiatry.2009.129

[B64] Skarupski KA Tangney CC Li H Evans DA Morris MC Mediterranean diet and depressive symptoms among older adults over time J Nutr Health Aging 2013 17 441 445 23636545 10.1007/s12603-012-0437-x PMC4454450

[B65] Rienks J Dobson AJ Mishra GD Mediterranean dietary pattern and prevalence and incidence of depressive symptoms in mid-aged women: results from a large community-based prospective study Eur J Clin Nutr 2013 67 75 82 23212131 10.1038/ejcn.2012.193

[B66] Lai JS Hiles S Bisquera A Hure AJ McEvoy M Attia J A systematic review and meta-analysis of dietary patterns and depression in community-dwelling adults Am J Clin Nutr 2014 99 181 197 24196402 10.3945/ajcn.113.069880

[B67] Psaltopoulou T Sergentanis TN Panagiotakos DB Sergentanis IN Kosti R Scarmeas N Mediterranean diet, stroke, cognitive impairment, and depression: a meta-analysis Ann Neurol 2013 74 580 591 23720230 10.1002/ana.23944

[B68] Nanri A Mizoue T Poudel-Tandukar K Noda M Kato M Kurotani K Goto A Oba S Inoue M Tsugane S Japan Public Health Center-based Prospective Study Group Dietary patterns and suicide in Japanese adults: the Japan Public Health Center-based Prospective Study Br J Psychiatry 2013 203 422 427 24115342 10.1192/bjp.bp.112.114793

[B69] Pham NM Nanri A Kurotani K Kuwahara K Kume A Sato M Hayabuchi H Mizoue T Green tea and coffee consumption is inversely associated with depressive symptoms in a Japanese working population Public Health Nutr 2014 17 625 633 A published erratum appears in *Public Health Nutr* 2014, 17:715 23453038 10.1017/S1368980013000360 PMC10282314

[B70] Jacka FN Ystrom E Brantsaeter AL Karevold E Roth C Haugen M Meltzer HM Schjolberg S Berk M Maternal and early postnatal nutrition and mental health of offspring by age 5 years: a prospective cohort study J Am Acad Child Adolesc Psychiatry 2013 52 1038 1047 24074470 10.1016/j.jaac.2013.07.002

[B71] Solfrizzi V Panza F Mediterranean diet and cognitive decline. A lesson from the whole-diet approach: what challenges lie ahead? J Alzheimers Dis 2014 39 283 286 24270209 10.3233/JAD-130831

[B72] Martínez-Lapiscina EH Clavero P Toledo E Estruch R Salas-Salvadó J San Julián B Sanchez-Tainta A Ros E Valls-Pedret C Martinez-Gonzalez MÁ Mediterranean diet improves cognition: the PREDIMED-NAVARRA randomised trial J Neurol Neurosurg Psychiatry 2013 84 1318 1325 23670794 10.1136/jnnp-2012-304792

[B73] Lang UE Borgwardt S Molecular mechanisms of depression: perspectives on new treatment strategies Cell Physiol Biochem 2013 31 761 777 23735822 10.1159/000350094

[B74] Yu Y Wang R Chen C Du X Ruan L Sun J Li J Zhang L O’Donnell JM Pan J Xu Y Antidepressant-like effect of *trans*-resveratrol in chronic stress model: behavioral and neurochemical evidences Psychiatr Res 2013 47 315 322 10.1016/j.jpsychires.2012.10.018 23174668

[B75] Pathak L Agrawal Y Dhir A Natural polyphenols in the management of major depression Expert Opin Investig Drugs 2013 22 863 880 10.1517/13543784.2013.794783 23642183

[B76] Sullivan EL Nousen EK Chamlou KA Maternal high fat diet consumption during the perinatal period programs offspring behavior Physiol Behav 2014 123 236 242 23085399 10.1016/j.physbeh.2012.07.014 PMC3594403

[B77] Sharma S Zhuang Y Gomez-Pinilla F High-fat diet transition reduces brain DHA levels associated with altered brain plasticity and behaviour Sci Rep 2012 2 431 22666534 10.1038/srep00431 PMC3362800

[B78] Fernandes C Grayton H Poston L Samuelsson AM Taylor PD Collier DA Rodriguez A Prenatal exposure to maternal obesity leads to hyperactivity in offspring Mol Psychiatry 2012 17 1159 1160 22158015 10.1038/mp.2011.164

[B79] Pase CS Roversi K Trevizol F Roversi K Kuhn FT Schuster AJ Vey LT Dias VT Barcelos RC Piccolo J Emanuelli T Bürger ME Influence of perinatal *trans* fat on behavioral responses and brain oxidative status of adolescent rats acutely exposed to stress Neuroscience 2013 247 242 252 23742847 10.1016/j.neuroscience.2013.05.053

[B80] Perveen T Hashmi BM Haider S Tabassum S Saleem S Siddiqui MA Role of monoaminergic system in the etiology of olive oil induced antidepressant and anxiolytic effects in rats ISRN Pharmacol 2013 2013 615685 23936669 10.1155/2013/615685 PMC3725699

[B81] Maric T Woodside B Luheshi GN The effects of dietary saturated fat on basal hypothalamic neuroinflammation in rats Brain Behav Immun 2014 36 35 45 24075847 10.1016/j.bbi.2013.09.011

[B82] Francis HM Stevenson RJ Higher reported saturated fat and refined sugar intake is associated with reduced hippocampal-dependent memory and sensitivity to interoceptive signals Behav Neurosci 2011 125 943 955 22023100 10.1037/a0025998

[B83] Papadopoulou A Markianos M Christodoulou C Lykouras L Plasma total cholesterol in psychiatric patients after a suicide attempt and in follow-up J Affect Disord 2013 148 440 443 23237826 10.1016/j.jad.2012.11.032

[B84] Almeida OP Yeap BB Hankey GJ Golledge J Flicker L HDL cholesterol and the risk of depression over 5 years Mol Psychiatry 2014 19 637 638 23999523 10.1038/mp.2013.113

[B85] Balanzá-Martínez V Fries GR Colpo GD Silveira PP Portella AK Tabarés-Seisdedos R Kapczinski F Therapeutic use of omega-3 fatty acids in bipolar disorder Expert Rev Neurother 2011 11 1029 1047 21721919 10.1586/ern.11.42

[B86] Matsuoka Y Nishi D Yonemoto N Hamazaki K Hashimoto K Hamazaki T Omega-3 fatty acids for secondary prevention of posttraumatic stress disorder after accidental injury: an open-label pilot study J Clin Psychopharmacol 2010 30 217 219 20520307 10.1097/JCP.0b013e3181d48830

[B87] Su KP Wang SM Pae CU Omega-3 polyunsaturated fatty acids for major depressive disorder Expert Opin Investig Drugs 2013 22 1519 1534 10.1517/13543784.2013.836487 24083675

[B88] Amminger GP Schäfer MR Papageorgiou K Klier CM Cotton SM Harrigan SM Mackinnon A McGorry PD Berger GE Long-chain ω-3 fatty acids for indicated prevention of psychotic disorders: a randomized, placebo-controlled trial Arch Gen Psychiatry 2010 67 146 154 20124114 10.1001/archgenpsychiatry.2009.192

[B89] Rucklidge JJ Johnstone J Kaplan BJ Magic bullet thinking–why do we continue to perpetuate this fallacy? Br J Psychiatry 2013 203 154 23908343 10.1192/bjp.203.2.154

[B90] Rucklidge JJ Kaplan BJ Broad-spectrum micronutrient formulas for the treatment of psychiatric symptoms: a systematic review Expert Rev Neurother 2013 13 49 73 23253391 10.1586/ern.12.143

[B91] Solati Z Jazayeri S Tehrani-Doost M Mahmoodianfard S Gohari MR Zinc monotherapy increases serum brain-derived neurotrophic factor (BDNF) levels and decreases depressive symptoms in overweight or obese subjects: a double-blind, randomized, placebo-controlled tria Nutr Neurosciin press. doi:10.1179/1476830513Y.0000000105 10.1179/1476830513Y.0000000105 24621065

[B92] Swardfager W Herrmann N Mazereeuw G Goldberger K Harimoto T Lanctôt KL Zinc in depression: a meta-analysis Biol Psychiatry 2013 74 872 878 23806573 10.1016/j.biopsych.2013.05.008

[B93] Młyniec K Budziszewska B Reczyński W Doboszewska U Pilc A Nowak G Zinc deficiency alters responsiveness to antidepressant drugs in mice Pharmacol Rep 2013 65 579 592 23950580 10.1016/s1734-1140(13)71035-1

[B94] Eder K Kirchgessner M Dietary fat influences the effect of zinc deficiency on liver lipids and fatty acids in rats force-fed equal quantities of diet J Nutr 1994 124 1917 1926 7931700 10.1093/jn/124.10.1917

[B95] Sadli N Ackland ML De Mel D Sinclair AJ Suphioglu C Effects of zinc and DHA on the epigenetic regulation of human neuronal cells Cell Physiol Biochem 2012 29 87 98 22415078 10.1159/000337590

[B96] Rucklidge JJ Frampton CM Gorman B Boggis A Vitamin-mineral treatment of ADHD in adults: a 1-year naturalistic follow-up of a randomized controlled trial J Atten Disordin press. doi:10.1177/1087054714530557 10.1177/1087054714530557 24804687

[B97] Pipingas A Camfield DA Stough C Cox KH Fogg E Tiplady B Sarris J White DJ Sali A Wetherell MA Scholey AB The effects of multivitamin supplementation on mood and general well-being in healthy young adults: a laboratory and at-home mobile phone assessment Appetite 2013 69 123 136 23727255 10.1016/j.appet.2013.05.016

[B98] Rucklidge JJ Harris AL Shaw IC Are the amounts of vitamins in commercially available dietary supplement formulations relevant for the management of psychiatric disorders in children? N Z Med J 2014 127 73 85 24806250

[B99] Dickinson A Blatman J El-Dash N Franco JC Consumer usage and reasons for using dietary supplements: report of a series of surveys J Am Coll Nutr 2014 33 176 182 24724775 10.1080/07315724.2013.875423

[B100] Davison KM Kaplan BJ Nutrient- and non-nutrient-based natural health product (NHP) use in adults with mood disorders: prevalence, characteristics and potential for exposure to adverse events BMC Complement Altern Med 2013 13 80 23570306 10.1186/1472-6882-13-80 PMC3626531

[B101] Astiz M Diz-Chaves Y Garcia-Segura LM Sub-chronic exposure to the insecticide dimethoate induces a proinflammatory status and enhances the neuroinflammatory response to bacterial lipopolysaccharide in the hippocampus and striatum of male mice Toxicol Appl Pharmacol 2013 272 263 271 23891859 10.1016/j.taap.2013.07.008

[B102] McCann D Barrett A Cooper A Crumpler D Dalen L Grimshaw K Kitchin E Lok K Porteous L Prince E Sonuga-Barke E Warner JO Stevenson J Food additives and hyperactive behaviour in 3-year-old and 8/9-year-old children in the community: a randomised, double-blinded, placebo-controlled trial Lancet 2007 370 1560 1567 A published erratum appears in *Lancet* 2007, **370**:1542 17825405 10.1016/S0140-6736(07)61306-3

[B103] Quines CB Rosa SG Da Rocha JT Gai BM Bortolatto CF Duarte MM Nogueira CW Monosodium glutamate, a food additive, induces depressive-like and anxiogenic-like behaviors in young rats Life Sci 2014 107 27 31 24802127 10.1016/j.lfs.2014.04.032

[B104] Cong WN Wang R Cai H Daimon CM Scheibye-Knudsen M Bohr VA Turkin R Wood WH 3rd Becker KG Moaddel R Maudsley S Martin B Long-term artificial sweetener acesulfame potassium treatment alters neurometabolic functions in C57BL/6 J mice PLoS One 2013 8 e70257 23950916 10.1371/journal.pone.0070257 PMC3737213

[B105] Peters SL Biesiekierski JR Yelland GW Muir JG Gibson PR Randomised clinical trial: gluten may cause depression in subjects with non-coeliac gluten sensitivity – an exploratory clinical study Aliment Pharmacol Ther 2014 39 1104 1112 24689456 10.1111/apt.12730

[B106] Prince M Patel V Saxena S Maj M Maselko J Phillips MR Rahman A No health without mental health Lancet 2007 370 859 877 17804063 10.1016/S0140-6736(07)61238-0

[B107] Gubata ME Urban N Cowan DN Niebuhr DW A prospective study of physical fitness, obesity, and the subsequent risk of mental disorders among healthy young adults in army training J Psychosom Res 2013 75 43 48 23751237 10.1016/j.jpsychores.2013.04.003

[B108] Sener U Ucok K Ulasli AM Genc A Karabacak H Coban NF Simsek H Cevik H Evaluation of health-related physical fitness parameters and association analysis with depression, anxiety, and quality of life in patients with fibromyalgia Int J Rheum Disin press. doi: 10.1111/1756-185X.12237 10.1111/1756-185X.12237 24289723

[B109] Searle A Calnan M Lewis G Campbell J Taylor A Turner K Patients’ views of physical activity as treatment for depression: a qualitative study Br J Gen Pract 2011 61 149 156 21439172 10.3399/bjgp11X567054 PMC3063043

[B110] Weinstein AA Deuster PA Francis JL Beadling C Kop WJ The role of depression in short-term mood and fatigue responses to acute exercise Int J Behav Med 2010 17 51 57 19333764 10.1007/s12529-009-9046-4

[B111] Ekkekakis P Parfitt G Petruzzello SJ The pleasure and displeasure people feel when they exercise at different intensities: decennial update and progress towards a tripartite rationale for exercise intensity prescription Sports Med 2011 41 641 671 21780850 10.2165/11590680-000000000-00000

[B112] Annesi J Relations of self-motivation, perceived physical condition, and exercise-induced changes in revitalization and exhaustion with attendance in women initiating a moderate cardiovascular exercise regimen Women Health 2005 42 77 93 16901889 10.1300/j013v42n03_05

[B113] Kwan BM Bryan A In-task and post-task affective response to exercise: translating exercise intentions into behaviour Br J Health Psychol 2010 15 115 131 19397847 10.1348/135910709X433267 PMC12240157

[B114] Huck CJ Johnston CS Beezhold BL Swan PD Vitamin C status and perception of effort during exercise in obese adults adhering to a calorie-reduced diet Nutrition 2013 29 42 45 22677357 10.1016/j.nut.2012.01.021

[B115] McMillan L Owen L Kras M Scholey A Behavioural effects of a 10-day Mediterranean diet: results from a pilot study evaluating mood and cognitive performance Appetite 2011 56 143 147 21115083 10.1016/j.appet.2010.11.149

[B116] Shahar DR Houston DK Hue TF Lee JS Sahyoun NR Tylavsky FA Geva D Vardi H Harris TB Adherence to Mediterranean diet and decline in walking speed over 8 years in community-dwelling older adults J Am Geriatr Soc 2012 60 1881 1888 23035758 10.1111/j.1532-5415.2012.04167.x PMC3470771

[B117] Zbeida M Goldsmith R Shimony T Vardi H Naggan L Shahar DR Mediterranean diet and functional indicators among older adults in non-Mediterranean and Mediterranean countries J Nutr Health Aging 2014 18 411 418 24676323 10.1007/s12603-014-0003-9 PMC12880335

[B118] Kivimäki M Shipley MJ Batty GD Hamer M Akbaraly TN Kumari M Jokela M Virtanen M Lowe GD Ebmeier KP Brunner EJ Singh-Manoux A Long-term inflammation increases risk of common mental disorder: a cohort study Mol Psychiatry 2014 19 149 150 23568195 10.1038/mp.2013.35 PMC3903110

[B119] Moylan S Maes M Wray NR Berk M The neuroprogressive nature of major depressive disorder: pathways to disease evolution and resistance, and therapeutic implications Mol Psychiatry 2013 18 595 606 22525486 10.1038/mp.2012.33

[B120] Marazziti D Rutigliano G Baroni S Landi P Dell’Osso L Metabolic syndrome and major depression CNS Spectrin press. doi:10.1017/S1092852913000667

[B121] Bested AC Logan AC Selhub EM Intestinal microbiota, probiotics and mental health: from Metchnikoff to modern advances: Part II – contemporary contextual research Gut Pathog 2013 5 3 23497633 10.1186/1757-4749-5-3 PMC3601973

[B122] Burnet PW Cowen PJ Psychobiotics highlight the pathways to happiness Biol Psychiatry 2013 74 708 709 24144322 10.1016/j.biopsych.2013.08.002

[B123] Ohland CL Kish L Bell H Thiesen A Hotte N Pankiv E Madsen KL Effects of *Lactobacillus helveticus* on murine behavior are dependent on diet and genotype and correlate with alterations in the gut microbiome Psychoneuroendocrinology 2013 38 1738 1747 23566632 10.1016/j.psyneuen.2013.02.008

[B124] Selhub EM Logan AC Bested AC Fermented foods, microbiota, and mental health: ancient practice meets nutritional psychiatry J Physiol Anthropol 2014 33 2 24422720 10.1186/1880-6805-33-2 PMC3904694

[B125] Pallebage-Gamarallage M Lam V Takechi R Galloway S Clark K Mamo J Restoration of dietary-fat induced blood–brain barrier dysfunction by anti-inflammatory lipid-modulating agents Lipids Health Dis 2012 11 117 22978403 10.1186/1476-511X-11-117 PMC3492058

[B126] Chang HC Tai YT Cherng YG Lin JW Liu SH Chen TL Chen RM Resveratrol attenuates high-fat diet-induced disruption of the blood–brain barrier and protects brain neurons from apoptotic insults J Agric Food Chem 2014 62 3466 3475 24694235 10.1021/jf403286w

[B127] Takechi R Pallebage-Gamarallage MM Lam V Giles C Mamo JC Nutraceutical agents with anti-inflammatory properties prevent dietary saturated-fat induced disturbances in blood–brain barrier function in wild-type mice J Neuroinflammation 2013 10 73 23782872 10.1186/1742-2094-10-73 PMC3693897

[B128] Rahe C Unrath M Berger K Dietary patterns and the risk of depression in adults: a systematic review of observational studies Eur J Nutr 2014 53 997 1013 24468939 10.1007/s00394-014-0652-9

[B129] Aneshensel CS Toward explaining mental health disparities J Health Soc Behav 2009 50 377 394 20099446 10.1177/002214650905000401

[B130] Wittayanukorn S Qian J Hansen RA Prevalence of depressive symptoms and predictors of treatment among U.S. adults from 2005 to 2010 Gen Hosp Psychiatry 2014 36 330 336 24462337 10.1016/j.genhosppsych.2013.12.009

[B131] Ban L Gibson JE West J Fiaschi L Oates MR Tata LJ Impact of socioeconomic deprivation on maternal perinatal mental illnesses presenting to UK general practice Br J Gen Pract 2012 62 e671 e678 23265226 10.3399/bjgp12X656801 PMC3459774

[B132] Rofey DL Kolko RP Iosif AM Silk JS Bost JE Feng W Szigethy EM Noll RB Ryan ND Dahl RE A longitudinal study of childhood depression and anxiety in relation to weight gain Child Psychiatry Hum Dev 2009 40 4 517 526 19404733 10.1007/s10578-009-0141-1 PMC2918233

[B133] Goodman E Whitaker RC A prospective study of the role of depression in the development and persistence of adolescent obesity Pediatrics 2002 110 497 504 12205250 10.1542/peds.110.3.497

[B134] Brumpton B Langhammer A Romundstad P Chen Y Mai XM The associations of anxiety and depression symptoms with weight change and incident obesity: the HUNT study Int J Obes 2013 37 1268 1274 10.1038/ijo.2012.204 23229732

[B135] Jeffery AN Hyland ME Hosking J Wilkin TJ Mood and its association with metabolic health in adolescents: a longitudinal study, EarlyBird 65 Pediatr Diabetesin press. doi:10.1111/pedi.12125 10.1111/pedi.12125 24552539

[B136] Brook JS Lee JY Brook DW Finch SJ Determinants of obesity: results from a longitudinal study of adolescents and adults living in an urban area Psychol Rep 2013 113 717 733 24693808 10.2466/15.13.PR0.113x26z6 PMC3979534

[B137] Anderson SE Cohen P Naumova EN Must A Association of depression and anxiety disorders with weight change in a prospective community-based study of children followed up into adulthood Arch Pediatr Adolesc Med 2006 160 285 291 16520448 10.1001/archpedi.160.3.285

[B138] Jacka FN Mykletun A Berk M Moving towards a population health approach to the primary prevention of common mental disorders BMC Med 2012 10 149 23186355 10.1186/1741-7015-10-149 PMC3534562

[B139] Rossen LM Neighbourhood economic deprivation explains racial/ethnic disparities in overweight and obesity among children and adolescents in the U.S.A J Epidemiol Community Health 2014 68 123 129 24072744 10.1136/jech-2012-202245 PMC5705001

[B140] Lindberg MA Dementieva Y Cavender J Why has the BMI gone up so drastically in the last 35 years? J Addict Med 2011 5 272 278 22107876 10.1097/ADM.0b013e3182118d41

[B141] Morgan D Sizemore GM Animal models of addiction: fat and sugar Curr Pharm Des 2011 17 1168 1172 21492084 10.2174/138161211795656747

[B142] Garber AK Lustig RH Is fast food addictive? Curr Drug Abuse Rev 2011 4 146 162 21999689 10.2174/1874473711104030146

[B143] Brooks RC Simpson SJ Raubenheimer D The price of protein: combining evolutionary and economic analysis to understand excessive energy consumption Obes Rev 2010 11 887 894 20230444 10.1111/j.1467-789X.2010.00733.x

[B144] Rao M Afshin A Singh G Mozaffarian D Do healthier foods and diet patterns cost more than less healthy options? A systematic review and meta-analysis BMJ Open 2013 3 e004277 10.1136/bmjopen-2013-004277 PMC3855594 24309174

[B145] Barosh L Friel S Engelhardt K Chan L The cost of a healthy and sustainable diet–who can afford it? Aust N Z J Public Health 2014 38 7 12 24494938 10.1111/1753-6405.12158

[B146] South T Holmes NM Martire SI Westbrook RF Morris MJ Rats eat a cafeteria-style diet to excess but eat smaller amounts and less frequently when tested with chow PLoS One 2014 9 e93506 24751610 10.1371/journal.pone.0093506 PMC3994000

[B147] Martire SI Maniam J South T Holmes N Westbrook RF Morris MJ Extended exposure to a palatable cafeteria diet alters gene expression in brain regions implicated in reward, and withdrawal from this diet alters gene expression in brain regions associated with stress Behav Brain Res 2014 265 132 141 24583192 10.1016/j.bbr.2014.02.027

[B148] Maniam J Morris MJ Palatable cafeteria diet ameliorates anxiety and depression-like symptoms following an adverse early environment Psychoneuroendocrinology 2010 35 717 728 19939573 10.1016/j.psyneuen.2009.10.013

[B149] Michels N Sioen I Braet C Huybrechts I Vanaelst B Wolters M De Henauw S Relation between salivary cortisol as stress biomarker and dietary pattern in children Psychoneuroendocrinology 2013 38 1512 1520 23332247 10.1016/j.psyneuen.2012.12.020

[B150] Weltens N Zhao D Van Oudenhove L Where is the comfort in comfort foods? Mechanisms linking fat signaling, reward, and emotion Neurogastroenterol Motil 2014 26 303 315 24548257 10.1111/nmo.12309

[B151] Tryon MS Carter CS Decant R Laugero KD Chronic stress exposure may affect the brain’s response to high calorie food cues and predispose to obesogenic eating habits Physiol Behav 2013 120 233 242 23954410 10.1016/j.physbeh.2013.08.010

[B152] Van Oudenhove L McKie S Lassman D Uddin B Paine P Coen S Gregory L Tack J Aziz Q Fatty acid-induced gut-brain signaling attenuates neural and behavioral effects of sad emotion in humans J Clin Invest 2011 121 3094 3099 21785220 10.1172/JCI46380 PMC3148741

[B153] Lee RE Heinrich KM Reese-Smith JY Regan GR Adamus-Leach HJ Obesogenic and youth oriented restaurant marketing in public housing neighborhoods Am J Health Behav 2014 38 218 224 24629550 10.5993/AJHB.38.2.7

[B154] Duran AC Diez Roux AV do Rosario do Latorre M Jaime PC Neighborhood socioeconomic characteristics and differences in the availability of healthy food stores and restaurants in Sao Paulo, Brazil Health Place 2013 23 39 47 23747923 10.1016/j.healthplace.2013.05.001 PMC3758426

[B155] DeVoe SE House J Zhong CB Fast food and financial impatience: a socioecological approach J Pers Soc Psychol 2013 105 476 494 23773044 10.1037/a0033484

[B156] Lowery BC Sloane DC The prevalence of harmful content on outdoor advertising in Los Angeles: land use, community characteristics, and the spatial inequality of a public health nuisance Am J Public Health 2014 104 658 664 24524512 10.2105/AJPH.2013.301694 PMC4025710

[B157] Lesser LI Zimmerman FJ Cohen DA Outdoor advertising, obesity, and soda consumption: a cross-sectional study BMC Public Health 2013 13 20 23305548 10.1186/1471-2458-13-20 PMC3556162

[B158] Yancey AK Cole BL Brown R Williams JD Hillier A Kline RS Ashe M Grier SA Backman D McCarthy WJ A cross-sectional prevalence study of ethnically targeted and general audience outdoor obesity-related advertising Milbank Q 2009 87 155 184 19298419 10.1111/j.1468-0009.2009.00551.x PMC2879171

[B159] Cohen DA Babey SH Contextual influences on eating behaviours: heuristic processing and dietary choices Obes Rev 2012 13 766 779 22551473 10.1111/j.1467-789X.2012.01001.x PMC3667220

[B160] Arredondo E Castaneda D Elder JP Slymen D Dozier D Brand name logo recognition of fast food and healthy food among children J Community Health 2009 34 73 78 18830690 10.1007/s10900-008-9119-3

[B161] Gardner MP Wansink B Kim J Park SB Better moods for better eating? How mood influences food choice J Consumer Psychol 2014 24 320 335

[B162] Pulcu E Trotter PD Thomas EJ McFarquhar M Juhasz G Sahakian BJ Deakin JF Zahn R Anderson IM Elliott R Temporal discounting in major depressive disorder Psychol Med 2014 44 1825 1834 10.1017/S0033291713002584 PMC4035754 24176142

[B163] Lu Q Tao F Hou F Zhang Z Sun Y Xu Y Xu S Zhao Y Cortisol reactivity, delay discounting and percent body fat in Chinese urban young adolescents Appetite 2014 72 13 20 24080189 10.1016/j.appet.2013.09.019

[B164] Irvine MA Worbe Y Bolton S Harrison NA Bullmore ET Voon V Impaired decisional impulsivity in pathological videogamers PLoS One 2013 8 e75914 24146789 10.1371/journal.pone.0075914 PMC3797823

[B165] Epstein LH Jankowiak N Fletcher KD Carr KA Nederkoorn C Raynor H Finkelstein E Women who are motivated to eat and discount the future are more obese Obesity 2014 22 1394 1399 24311480 10.1002/oby.20661 PMC4007365

[B166] Garza KB Harris CV Bolding MS Examination of value of the future and health beliefs to explain dietary and physical activity behaviors Res Social Adm Pharm 2013 9 851 862 23287815 10.1016/j.sapharm.2012.12.001

[B167] van der Wal AJ Schade HM Krabbendam L van Vugt M Do natural landscapes reduce future discounting in humans? Proc Biol Sci 2013 280 20132295 24197412 10.1098/rspb.2013.2295 PMC3826228

[B168] Zimmerman FJ Shimoga SV The effects of food advertising and cognitive load on food choices BMC Public Health 2014 14 342 24721289 10.1186/1471-2458-14-342 PMC4021209

[B169] Laxer RE Janssen I The proportion of excessive fast-food consumption attributable to the neighborhood food environment among youth living within 1 km of their school Appl Physiol Nutr Metab 2014 39 480 486 24669990 10.1139/apnm-2013-0208

[B170] Jago R Baranowski T Baranowski JC Cullen KW Thompson D Distance to food stores & adolescent male fruit and vegetable consumption: mediation effects Int J Behav Nutr Phys Act 2007 4 35 17850673 10.1186/1479-5868-4-35 PMC2014759

[B171] Timperio A Ball K Roberts R Campbell K Andrianopoulos N Crawford D Children’s fruit and vegetable intake: associations with the neighbourhood food environment Prev Med 2008 46 331 335 18164753 10.1016/j.ypmed.2007.11.011

[B172] Nicklett EJ Szanton S Sun K Ferrucci L Fried LP Guralnik JM Semba RD Neighborhood socioeconomic status is associated with serum carotenoid concentrations in older, community-dwelling women J Nutr 2011 141 284 289 21178091 10.3945/jn.110.129684 PMC3021448

[B173] Stimpson JP Nash AC Ju H Eschbach K Neighborhood deprivation is associated with lower levels of serum carotenoids among adults participating in the third national health and nutrition examination survey J Am Diet Assoc 2007 107 1895 1902 17964308 10.1016/j.jada.2007.08.016

[B174] Twenge JM Does online social media lead to social connection or social disconnection? J Coll Char 2013 14 11 20

[B175] Chen W Lee KH Sharing, liking, commenting, and distressed? The pathway between Facebook interaction and psychological distress Cyberpsychol Behav Soc Netw 2013 16 728 734 23745614 10.1089/cyber.2012.0272

[B176] Kross E Verduyn P Demiralp E Park J Lee DS Lin N Shablack H Jonides J Ybarra O Facebook use predicts declines in subjective well-being in young adults PLoS One 2013 8 e69841 23967061 10.1371/journal.pone.0069841 PMC3743827

[B177] Becker MW Alzahabi R Hopwood CJ Media multitasking is associated with symptoms of depression and social anxiety Cyberpsychol Behav Soc Netw 2013 16 132 135 23126438 10.1089/cyber.2012.0291

[B178] Primack B Swanier B Georgiopoulos AM Land SR Fine MJ Association between media use in adolescence and depression in young adulthood Arch Gen Psychiatry 2009 66 181 188 19188540 10.1001/archgenpsychiatry.2008.532 PMC3004674

[B179] Van den Eijnden R Meerkerk GJ Vermulst AA Spijkerman R Engels RC Online communication, compulsive internet use, and psychological well-being among adolescents: a longitudinal study Dev Psychol 2008 44 655 665 18473634 10.1037/0012-1649.44.3.655

[B180] Salanova M Llorens S Cifre E The dark side of technologies: technostress among users of information and communication technologies Int J Psychol 2013 48 422 436 22731610 10.1080/00207594.2012.680460

[B181] Lucas M Mekary R Pan A Mirzaei F O’Reilly EJ Willett WC Koenen K Okereke OI Ascherio A Relation between clinical depression risk and physical activity and time spent watching television in older women: a 10-year prospective follow-up study Am J Epidemiol 2011 174 1017 1027 21984659 10.1093/aje/kwr218 PMC3243936

[B182] Misra S Stokols D Psychological and health outcomes of perceived information overload Environ Behav 2012 44 737 759

[B183] Ding D Sugiyama T Winkler E Cerin E Wijndaele K Owen N Correlates of change in adults’ television viewing time: a four-year follow-up study Med Sci Sports Exerc 2012 44 1287 1292 22297804 10.1249/MSS.0b013e31824ba87e

[B184] Sugiyama T Salmon J Dunstan DW Bauman AE Owen N Neighborhood walkability and TV viewing time among Australian adults Am J Prev Med 2007 33 444 449 18022059 10.1016/j.amepre.2007.07.035

[B185] Salmon J Veitch J Abbott G ChinAPaw M Brug JJ te Velde SJ Cleland V Hume C Crawford D Ball K Are associations between the perceived home and neighbourhood environment and children’s physical activity and sedentary behaviour moderated by urban/rural location? Health Place 2013 24 44 53 24036187 10.1016/j.healthplace.2013.07.010

[B186] Tandon PS Zhou C Sallis JF Cain KL Frank LD Saelens BE Home environment relationships with children’s physical activity, sedentary time, and screen time by socioeconomic status Int J Behav Nutr Phys Act 2012 9 88 22835155 10.1186/1479-5868-9-88 PMC3413573

[B187] Kremer P Elshaug C Leslie E Toumbourou JW Patton GC Williams J Physical activity, leisure-time screen use and depression among children and young adolescents J Sci Med Sport 2014 17 183 187 23648221 10.1016/j.jsams.2013.03.012

[B188] Al-Hazzaa HM Al-Sobayel HI Abahussain NA Qahwaji DM Alahmadi MA Musaiger AO Association of dietary habits with levels of physical activity and screen time among adolescents living in Saudi Arabia J Hum Nutr Diet 2014 27 Suppl 2 204 213 23889093 10.1111/jhn.12147

[B189] Olafsdottir S Berg C Eiben G Lanfer A Reisch L Ahrens W Kourides Y Molnár D Moreno LA Siani A Veidebaum T Lissner L Young children’s screen activities, sweet drink consumption and anthropometry: results from a prospective European study Eur J Clin Nutr 2014 68 223 228 24253759 10.1038/ejcn.2013.234

[B190] Gebremariam MK Bergh IH Andersen LF Ommundsen Y Totland TH Bjelland M Grydeland M Lien N Are screen-based sedentary behaviors longitudinally associated with dietary behaviors and leisure-time physical activity in the transition into adolescence? Int J Behav Nutr Phys Act 2013 10 9 23351357 10.1186/1479-5868-10-9 PMC3560151

[B191] Barr-Anderson DJ Larson NI Nelson MC Neumark-Sztainer D Story M Does television viewing predict dietary intake five years later in high school students and young adults? Int J Behav Nutr Phys Act 2009 6 7 19183442 10.1186/1479-5868-6-7 PMC2643350

[B192] Lyons EJ Tate DF Ward DS The better the story, the bigger the serving: narrative transportation increases snacking during screen time in a randomized trial Int J Behav Nutr Phys Act 2013 10 60 23680389 10.1186/1479-5868-10-60 PMC3660271

[B193] Mellecker RR Lanningham-Foster L Levine JA McManus AM Energy intake during activity enhanced video game play Appetite 2010 55 343 347 20655345 10.1016/j.appet.2010.07.008

[B194] Mink M Evans A Moore CG Calderon KS Deger S Nutritional imbalance endorsed by televised food advertisements J Am Diet Assoc 2010 110 904 910 20497780 10.1016/j.jada.2010.03.020

[B195] Bragg MA Yanamadala S Roberto CA Harris JL Brownell KD Athlete endorsements in food marketing Pediatrics 2013 132 805 810 24101762 10.1542/peds.2013-0093

[B196] Wood B Rea MS Plitnick B Figueiro MG Light level and duration of exposure determine the impact of self-luminous tablets on melatonin suppression Appl Ergon 2013 44 237 240 22850476 10.1016/j.apergo.2012.07.008

[B197] Peuhkuri K Sihvola N Korpela R Diet promotes sleep duration and quality Nutr Res 2012 32 309 319 22652369 10.1016/j.nutres.2012.03.009

[B198] St-Onge MP Wolfe S Sy M Shechter A Hirsch J Sleep restriction increases the neuronal response to unhealthy food in normal-weight individuals Int J Obes 2014 38 411 416 10.1038/ijo.2013.114 PMC3883872 23779051

[B199] Fonken LK Lieberman RA Weil ZM Nelson RJ Dim light at night exaggerates weight gain and inflammation associated with a high-fat diet in male mice Endocrinology 2013 154 3817 3825 23861373 10.1210/en.2013-1121

[B200] Summa KC Voigt RM Forsyth CB Shaikh M Cavanaugh K Tang Y Vitaterna MH Song S Turek FW Keshavarzian A Disruption of the circadian clock in mice increases intestinal permeability and promotes alcohol-induced hepatic pathology and inflammation PLoS One 2013 8 e67102 23825629 10.1371/journal.pone.0067102 PMC3688973

[B201] Voigt RM Forsyth CB Green SJ Mutlu E Engen P Vitaterna MH Turek FW Keshavarzian A Circadian disorganization alters intestinal microbiota PLoS One 2014 9 e97500 24848969 10.1371/journal.pone.0097500 PMC4029760

[B202] Hendy HM Which comes first in food–mood relationships, foods or moods? Appetite 2012 58 771 775 22123609 10.1016/j.appet.2011.11.014

[B203] Kimura Y Wada T Okumiya K Ishimoto Y Fukutomi E Kasahara Y Chen W Sakamoto R Fujisawa M Otsuka K Matsubayashi K Eating alone among community-dwelling Japanese elderly: association with depression and food diversity J Nutr Health Aging 2012 16 728 731 23076516 10.1007/s12603-012-0067-3 PMC12878549

[B204] Nicklett EJ Semba RD Simonsick EM Szanton S Bandeen-Roche K Ferrucci L Guralnik JM Fried LP Diet quality and social support: factors associated with serum carotenoid concentrations among older disabled women (the Women’s Health and Aging Study) J Nutr Health Aging 2012 16 511 518 22659988 10.1007/s12603-012-0031-2 PMC3475721

[B205] United Nations Educational, Scientific and Cultural Organization (UNESCO) Lists of Intangible Cultural Heritage in Need of Urgent Safeguarding and Register of Best Safeguarding Practices [http://www.unesco.org/culture/ich/index.php?pg=00559] (accessed 21 July 2014)

[B206] Morinaka T Wozniewicz M Jeszka J Bajerska J Nowaczyk P Sone Y Westernization of dietary patterns among young Japanese and polish females – a comparison study Ann Agric Environ Med 2013 20 122 130 23540225

[B207] Serra-Majem L Bach-Faig A Raidó-Quintana B Nutritional and cultural aspects of the Mediterranean diet Int J Vitam Nutr Res 2012 82 157 162 23258395 10.1024/0300-9831/a000106

[B208] Grosso G Marventano S Giorgianni G Raciti T Galvano F Mistretta A Mediterranean diet adherence rates in Sicily, southern Italy Public Health Nutrin press, doi:10.1017/S1368980013002188 10.1017/S1368980013002188 PMC11108722 23941897

[B209] Grosso G Marventano S Buscemi S Scuderi A Matalone M Platania A Giorgianni G Rametta S Nolfo F Galvano F Mistretta A Factors associated with adherence to the Mediterranean diet among adolescents living in Sicily, Southern Italy Nutrients 2013 5 4908 4923 24304608 10.3390/nu5124908 PMC3875926

[B210] Farajian P Risvas G Karasouli K Pounis GD Kastorini CM Panagiotakos DB Zampelas A Very high childhood obesity prevalence and low adherence rates to the Mediterranean diet in Greek children: the GRECO study Atherosclerosis 2011 217 525 530 21561621 10.1016/j.atherosclerosis.2011.04.003

[B211] Roccaldo R Censi L D’Addezio L Toti E Martone D D’Addesa D Cernigliaro A the ZOOM8 Study group Adherence to the Mediterranean diet in Italian school children (The ZOOM8 Study) Int J Food Sci Nutr 2014 65 621 628 24527679 10.3109/09637486.2013.873887

[B212] Motohashi K Kaneko Y Fujita K Motohashi Y Nakamura A Interest in dietary pattern, social capital, and psychological distress: a cross-sectional study in a rural Japanese community BMC Public Health 2013 13 933 24099097 10.1186/1471-2458-13-933 PMC3851249

[B213] Konner M Eaton SB Paleolithic nutrition: twenty-five years later Nutr Clin Pract 2010 25 594 602 21139123 10.1177/0884533610385702

[B214] Armelagos GJ Brain evolution, the determinates of food choice, and the omnivore’s dilemma Crit Rev Food Sci Nutr 2014 54 1330 1341 24564590 10.1080/10408398.2011.635817

[B215] Lucock MD Martin CE Yates ZR Veysey M Diet and our genetic legacy in the recent anthropocene: a Darwinian perspective to nutritional health J Evid Based Complem Altern Med 2014 19 68 83 10.1177/2156587213503345 24647381

[B216] Varga S Evolutionary psychiatry and depression: testing two hypotheses Med Health Care Philos 2012 15 41 52 21221814 10.1007/s11019-010-9305-9

[B217] Stearns SC Evolutionary medicine: its scope, interest and potential Proc Biol Sci 2012 279 4305 4321 22933370 10.1098/rspb.2012.1326 PMC3479795

[B218] Ruiz-Núñez B Pruimboom L Dijck-Brouwer DA Muskiet FA Lifestyle and nutritional imbalances associated with Western diseases: causes and consequences of chronic systemic low-grade inflammation in an evolutionary context J Nutr Biochem 2013 24 1183 1201 23657158 10.1016/j.jnutbio.2013.02.009

[B219] Bilbo SD Schwatrz JM The immune system and developmental programming of brain and behavior Front Neurosci 2012 33 267 286 10.1016/j.yfrne.2012.08.006 PMC3484177 22982535

[B220] Bendiks M Kopp MV The relationship between advances in understanding the microbiome and the maturing hygiene hypothesis Curr Allergy Asthma Rep 2013 13 487 494 23934550 10.1007/s11882-013-0382-8

[B221] Kondrashova A Seiskari T Ilonen J Knip M Hyöty H The ‘Hygiene hypothesis’ and the sharp gradient in the incidence of autoimmune and allergic diseases between Russian Karelia and Finland APMIS 2013 121 478 493 23127244 10.1111/apm.12023

[B222] Frei R Lauener RP Crameri R O’Mahony L Microbiota and dietary interactions–an update to the hygiene hypothesis? Allergy 2012 67 451 461 22257145 10.1111/j.1398-9995.2011.02783.x

[B223] The Editors The editors notebook Med Standard 1922 45 20

[B224] Stefansson V Food of the ancient and modern Stone Age man J Am Diet Assoc 1937 13 102 119

[B225] Oakes EH Encyclopedia of World Scientists. Revised edition 2007 New York: Facts on File/Infobase Publishing

[B226] Stern F The nutritionist looks at mental hygiene Mental Hyg J 1930 14 54 66

[B227] Eaton SB Konner M Shostak M Stone Agers in the fast lane: chronic degenerative diseases in evolutionary perspective Am J Med 1988 84 739 749 3135745 10.1016/0002-9343(88)90113-1

[B228] Wu X Zhang C Goldberg P Cohen D Pan Y Arpin T Bar-Yosef O Early pottery at 20,000 years ago in Xianrendong Cave, China Science 2012 336 1696 1700 22745428 10.1126/science.1218643

[B229] Speth J Boiling vs. roasting in the Paleolithic: broadening the “broadening food spectrum” J Isr Prehistor Soc 2010 40 63 83

[B230] Zielinski L Stone age stew? Soup making may be older than we’d thought [http://www.npr.org/blogs/thesalt/2013/02/06/171104410/stone-age-stew-soup-making-may-be-older-than-wed-thought] (accessed 19 July 2014)

[B231] Seligman L The history of Japanese cuisine Japan Quart 1994 41 165 180

[B232] Kuroda M Nozawa Y Effect of dried-bonito broth on mood states: a pooled analysis of four randomized controlled human trials Biomed Res 2008 29 175 179 18724004 10.2220/biomedres.29.175

[B233] Funatsu S Kondoh T Kawase T Ikeda H Nagasawa M Denbow DM Furuse M Long-term consumption of dried bonito *dashi* (a traditional Japanese fish stock) reduces anxiety and modifies central amino acid levels in rats Nutr Neurosciin press. doi:10.1179/1476830514Y.0000000124 10.1179/1476830514Y.0000000124 24701973

[B234] Uribarri J Woodruff S Goodman S Cai W Chen X Pyzik R Yong A Striker GE Vlassara H Advanced glycation end products in foods and a practical guide to their reduction in the diet J Am Diet Assoc 2010 110 911 916.e12 20497781 10.1016/j.jada.2010.03.018 PMC3704564

[B235] Luévano-Contreras C Garay-Sevilla ME Wrobel K Malacara JM Wrobel K Dietary advanced glycation end products restriction diminishes inflammation markers and oxidative stress in patients with type 2 diabetes mellitus J Clin Biochem Nutr 2013 52 22 26 23341693 10.3164/jcbn.12-40 PMC3541414

[B236] Uribarri J Stirban A Sander D Cai W Negrean M Buenting CE Koschinsky T Vlassara H Single oral challenge by advanced glycation end products acutely impairs endothelial function in diabetic and nondiabetic subjects Diabetes Care 2007 30 2579 2582 17496238 10.2337/dc07-0320

[B237] Mark AB Poulsen MW Andersen S Andersen JM Bak MJ Ritz C Holst JJ Nielsen J de Courten B Dragsted LO Bügel SG Consumption of a diet low in advanced glycation end products for 4 weeks improves insulin sensitivity in overweight women Diabetes Care 2014 37 88 95 23959566 10.2337/dc13-0842

[B238] Vlassara H Uribarri J Advanced glycation end products (AGE) and diabetes: cause, effect, or both? Curr Diab Rep 2014 14 453 24292971 10.1007/s11892-013-0453-1 PMC3903318

[B239] Emanuele E Martinelli V Carlin MV Fugazza E Barale F Politi P Serum levels of soluble receptor for advanced glycation endproducts (sRAGE) in patients with different psychiatric disorders Neurosci Lett 2011 487 99 102 20937362 10.1016/j.neulet.2010.10.003

[B240] Chen G Wu Y Wang T Liang J Lin W Li L Wen J Lin L Huang H Association between serum endogenous secretory receptor for advanced glycation end products and risk of type 2 diabetes mellitus with combined depression in the Chinese population Diabetes Technol Ther 2012 14 936 942 22856651 10.1089/dia.2012.0072 PMC3458998

[B241] Kamel KS Schreiber M Halperin ML Integration of the response to a dietary potassium load: a Paleolithic perspective Nephrol Dial Transplant 2014 29 982 989 24789504 10.1093/ndt/gft499

[B242] Ströhle A Hahn A Sebastian A Latitude, local ecology, and hunter-gatherer dietary acid load: implications from evolutionary ecology Am J Clin Nutr 2010 92 940 945 20702605 10.3945/ajcn.2010.29815

[B243] Frassetto L Morris RC Jr Sellmeyer DE Todd K Sebastian A Diet, evolution and aging–the pathophysiologic effects of the post-agricultural inversion of the potassium-to-sodium and base-to-chloride ratios in the human diet Eur J Nutr 2001 40 200 213 11842945 10.1007/s394-001-8347-4

[B244] Fagherazzi G Vilier A Bonnet F Lajous M Balkau B Boutron-Rualt MC Clavel-Chapelon F Dietary acid load and risk of type 2 diabetes: the E3N-EPIC cohort study Diabetologia 2014 57 313 320 24232975 10.1007/s00125-013-3100-0

[B245] Murakami K Sasaki S Takahashi Y Uenishi K Association between dietary acid–base load and cardiometabolic risk factors in young Japanese women Br J Nutr 2008 100 642 651 18279559 10.1017/S0007114508901288

[B246] Remer T Berkemeyer S Rylander R Vormann J Muscularity and adiposity in addition to net acid excretion as predictors of 24-h urinary pH in young adults and elderly Eur J Clin Nutr 2007 61 605 609 17119545 10.1038/sj.ejcn.1602560

[B247] Dawson-Hughes B Harris SS Ceglia L Alkaline diets favor lean tissue mass in older adults Am J Clin Nutr 2008 87 662 665 18326605 10.1093/ajcn/87.3.662 PMC2597402

[B248] Berkemeyer S Acid–base balance and weight gain: are there crucial links via protein and organic acids in understanding obesity? Med Hypotheses 2009 73 347 356 19410381 10.1016/j.mehy.2008.09.059

[B249] Torres SJ Nowson CA Worsley A Dietary electrolytes are related to mood Br J Nutr 2008 100 1038 1045 18466657 10.1017/S0007114508959201

[B250] Maurer M Riesen W Muser J Hutler HN Krapf R Neutralization of Western diet inhibits bone resorption independently of K intake and reduces cortisol secretion in humans Am J Physiol Renal Physiol 2003 284 F32 F40 12388390 10.1152/ajprenal.00212.2002

[B251] Epel E Psychological and metabolic stress: a recipe for accelerated cellular aging? Hormones 2009 8 7 22 19269917 10.14310/horm.2002.1217

[B252] Robey IF Examining the relationship between diet-induced acidosis and cancer Nutr Metab 2012 9 72 10.1186/1743-7075-9-72 PMC3571898 22853725

[B253] Robey IF Baggett BK Kirkpatrick ND Roe DJ Dosescu J Sloane BF Hashim AI Morse DL Raghunand N Gatenby RA Gillies RJ Bicarbonate increases tumor pH and inhibits spontaneous metastases Cancer Res 2009 69 2260 2268 19276390 10.1158/0008-5472.CAN-07-5575 PMC2834485

[B254] Amodu A Abramowitz MK Dietary acid, age, and serum bicarbonate levels among adults in the United States Clin J Am Soc Nephrol 2013 8 2034 2042 24052219 10.2215/CJN.03600413 PMC3848400

[B255] Ziemann AE Allen JE Dahdaleh NS Drebot II Coryell MW Wunsch AM Lynch CM Faraci FM Howard MA 3rd Welsh MJ Wemmie JA The amygdala is a chemosensor that detects carbon dioxide and acidosis to elicit fear behavior Cell 2009 139 1012 1021 19945383 10.1016/j.cell.2009.10.029 PMC2808123

[B256] Coryell MW Wunsch AM Haenfler JM Allen JE Schnizler M Ziemann AE Cook MN Dunning JP Price MP Rainier JD Liu Z Light AR Langbehn DR Wemmie JA Acid-sensing ion channel-1a in the amygdala, a novel therapeutic target in depression-related behavior J Neurosci 2009 29 5381 5388 19403806 10.1523/JNEUROSCI.0360-09.2009 PMC2710967

[B257] Davison KM Kaplan BJ Nutrient intakes are correlated with overall psychiatric functioning in adults with mood disorders Can J Psychiatry 2012 57 85 92 22340148 10.1177/070674371205700205

[B258] Loftfield E Yi S Curtis CJ Bartley K Kansagra SM Potassium and fruit and vegetable intakes in relation to social determinants and access to produce in New York City Am J Clin Nutr 2013 98 1282 1288 24025631 10.3945/ajcn.113.059204

[B259] Masento NA Golightly M Field DT Butler LT van Reekum CM Effects of hydration status on cognitive performance and mood Br J Nutr 2014 111 1841 1852 24480458 10.1017/S0007114513004455

[B260] Strachan DP Hay fever, hygiene, and household size BMJ 1989 299 1259 1260 2513902 10.1136/bmj.299.6710.1259 PMC1838109

[B261] Wold AE The hygiene hypothesis revised: is the rising frequency of allergy due to changes in the intestinal flora? Allergy 1998 53 46 Suppl 20 25 9825991 10.1111/j.1398-9995.1998.tb04953.x

[B262] Logan AC Venket Rao A Irani D Chronic fatigue syndrome: lactic acid bacteria may be of therapeutic value Med Hypotheses 2003 60 915 923 12699726 10.1016/s0306-9877(03)00096-3

[B263] Logan AC Katzman M Major depressive disorder: probiotics may be an adjuvant therapy Med Hypotheses 2005 64 533 538 15617861 10.1016/j.mehy.2004.08.019

[B264] Schnorr SL Candela M Rampelli S Centanni M Consolandi C Basaglia G Turroni S Biagi E Peano C Severgnini M Fiori J Gotti R De Bellis G Luiselli D Brigidi P Mabulla A Marlowe F Henry AG Crittenden AN Gut microbiome of the Hadza hunter-gatherers Nat Commun 2014 5 3654 24736369 10.1038/ncomms4654 PMC3996546

[B265] De Filippo C Cavalieri D Di Paola M Ramazzotti M Poullet JB Massart S Collini S Pieraccini G Lionetti P Impact of diet in shaping gut microbiota revealed by a comparative study in children from Europe and rural Africa Proc Natl Acad Sci U S A 2010 107 14691 14696 20679230 10.1073/pnas.1005963107 PMC2930426

[B266] Yatsunenko T Rey FE Manary MJ Trehan I Dominguez-Bello MG Contreras M Magris M Hidalgo G Baldassano RN Anokhin AP Heath AC Warner B Reeder J Kuczynski J Caporaso JG Lozupone CA Lauber C Clemente JC Knights D Knight R Gordon JI Human gut microbiome viewed across age and geography Nature 2012 486 222 227 22699611 10.1038/nature11053 PMC3376388

[B267] Tyakht AV Kostryukova ES Popenko AS Belenikin MS Pavlenko AV Larin AK Karpova IY Selezneva OV Semashko TA Ospanova EA Babenko VV Maev IV Cheremushkin SV Kucheryavyy YA Shcherbakov PL Grinevich VB Efimov OI Sas EI Abdulkhakov RA Abdulkhakov SR Lyalyukova EA Livzan MA Vlassov VV Sagdeev RZ Tsukanov VV Osipenko MF Kozlova IV Tkachev AV Sergienko VI Alexeev DG Govorun VM Human gut microbiota community structures in urban and rural populations in Russia Nat Commun 2013 4 2469 24036685 10.1038/ncomms3469 PMC3778515

[B268] Tito RY Knights D Metcalf J Obregon-Tito AJ Cleeland L Najar F Roe B Reinhard K Sobolik K Belknap S Foster M Spicer P Knight R Lewis CM Jr Insights from characterizing extinct human gut microbiomes PLoS One 2012 7 e51146 23251439 10.1371/journal.pone.0051146 PMC3521025

[B269] Spreadbury I Comparison with ancestral diets suggests dense acellular carbohydrates promote an inflammatory microbiota, and may be the primary dietary cause of leptin resistance and obesity Diabetes Metab Syndr Obes 2012 5 175 189 22826636 10.2147/DMSO.S33473 PMC3402009

[B270] Kelishadi R Poursafa P A review on the genetic, environmental, and lifestyle aspects of the early-life origins of cardiovascular disease Curr Probl Pediatr Adolesc Health Care 2014 44 54 72 24607261 10.1016/j.cppeds.2013.12.005

[B271] Benyshek DC The “early life” origins of obesity-related health disorders: new discoveries regarding the intergenerational transmission of developmentally programmed traits in the global cardiometabolic health crisis Am J Phys Anthropol 2013 152 Suppl 57 79 93 24249592 10.1002/ajpa.22393

[B272] Zhang R Lu J Kong X Jin L Luo C Targeting epigenetics in nervous system disease CNS Neurol Disord Drug Targets 2013 12 126 141 23244434 10.2174/1871527311312010018

[B273] Kofink D Boks MP Timmers HT Kas MJ Epigenetic dynamics in psychiatric disorders: environmental programming of neurodevelopmental processes Neurosci Biobehav Rev 2013 37 831 845 23567520 10.1016/j.neubiorev.2013.03.020

[B274] Dauncey NJ Genomic and epigenomic insights into nutrition and brain disorders Nutrients 2013 5 887 914 23503168 10.3390/nu5030887 PMC3705325

[B275] Rogers CE Lenze SN Luby JL Late preterm birth, maternal depression, and risk of preschool psychiatric disorders J Am Acad Child Adolesc Psychiatry 2013 52 309 318 23452687 10.1016/j.jaac.2012.12.005 PMC3589137

[B276] Grieger JA Grzeskowiak LE Clifton VL Preconception dietary patterns in human pregnancies are associated with preterm delivery J Nutr 2014 144 1075 1080 24790026 10.3945/jn.114.190686

[B277] Englund-Ögge L Brantsæter AL Sengpiel V Haugen M Birgisdottir BE Myhre R Meltzer HM Jacobsson B Maternal dietary patterns and preterm delivery: results from large prospective cohort study BMJ 2014 348 g1446 24609054 10.1136/bmj.g1446 PMC3942565

[B278] Julvez J Torrent M Guxens M Antó JM Guerra S Sunyer J Neuropsychologic status at the age 4 years and atopy in a population-based birth cohort Allergy 2009 64 1279 1285 19236318 10.1111/j.1398-9995.2009.01987.x

[B279] Calam R Gregg L Simpson A Simpson B Woodcock A Custovic A Behavior problems antecede the development of wheeze in childhood: a birth cohort study Am J Respir Crit Care Med 2005 171 323 327 15557132 10.1164/rccm.200406-791OC

[B280] Brüne M Hochberg Z Secular trends in new childhood epidemics: insights from evolutionary medicine BMC Med 2013 11 226 24228767 10.1186/1741-7015-11-226 PMC3852731

[B281] Jacka FN Reavley NJ Jorm AF Toumbourou JW Lewis AJ Berk M Prevention of common mental disorders: what can we learn from those who have gone before and where do we go next? Aust N Z J Psychiatry 2013 47 920 929 23798717 10.1177/0004867413493523

[B282] Ajdacic-Gross V The prevention of mental disorders has a bright future Front Public Health 2014 2 60 24926477 10.3389/fpubh.2014.00060 PMC4044585

[B283] Goldney RD Eckert KA Hawthorne G Taylor AW Changes in the prevalence of major depression in an Australian community sample between 1998 and 2008 Aust N Z J Psychiatry 2010 44 901 910 20932204 10.3109/00048674.2010.490520

[B284] Su KP The biological mechanism of antidepressant effect of omega-3 fatty acids: how does fish oil act as a “mind-body interface?” NeuroSignals 2009 17 144 152 19190401 10.1159/000198167

[B285] Sánchez-Villegas A Toledo E de Irala J Ruiz-Canela M Pla-Vidal J Martínez-González MA Fast-food and commercial baked goods consumption and the risk of depression Public Health Nutr 2012 15 424 432 21835082 10.1017/S1368980011001856

[B286] Crawford GB Khedkar A Flaws JA Sorkin JD Gallicchio L Depressive symptoms and self-reported fast-food intake in midlife women Prev Med 2011 52 254 257 21276813 10.1016/j.ypmed.2011.01.006 PMC3062726

[B287] Hirth JM Rahman M Berenson AB The association of posttraumatic stress disorder with fast food and soda consumption and unhealthy weight loss behaviors among young women J Womens Health 2011 20 1141 1149 10.1089/jwh.2010.2675 PMC3153863 21751875

